# The Role of Ionic Liquids in the Pharmaceutical Field: An Overview of Relevant Applications

**DOI:** 10.3390/ijms21218298

**Published:** 2020-11-05

**Authors:** Sónia N. Pedro, Carmen S. R. Freire, Armando J. D. Silvestre, Mara G. Freire

**Affiliations:** Department of Chemistry, CICECO-Aveiro Institute of Materials, University of Aveiro, 3810-193 Aveiro, Portugal; soniapedro@ua.pt (S.N.P.); cfreire@ua.pt (C.S.R.F.); armsil@ua.pt (A.J.D.S.)

**Keywords:** active pharmaceutical ingredients, drug delivery systems, formulations, ionic liquids, solubility, permeability

## Abstract

Solubility, bioavailability, permeation, polymorphism, and stability concerns associated to solid-state pharmaceuticals demand for effective solutions. To overcome some of these drawbacks, ionic liquids (ILs) have been investigated as solvents, reagents, and anti-solvents in the synthesis and crystallization of active pharmaceutical ingredients (APIs), as solvents, co-solvents and emulsifiers in drug formulations, as pharmaceuticals (API-ILs) aiming liquid therapeutics, and in the development and/or improvement of drug-delivery-based systems. The present review focuses on the use of ILs in the pharmaceutical field, covering their multiple applications from pharmaceutical synthesis to drug delivery. The most relevant research conducted up to date is presented and discussed, together with a critical analysis of the most significant IL-based strategies in order to improve the performance of therapeutics and drug delivery systems.

## 1. Introduction

Pharmaceuticals play a major role in medical care, boosting life quality and expectancy, especially when considering chronic diseases [1]. The global prescription of medicines is forecast to grow up to nearly $1.2 trillion by 2022 [2]. Although active pharmaceutical ingredients (APIs) can be commercialized in several dosage forms, crystalline forms have been the preferred option [3,4]. However, 40 to 70% of the drugs under development present low water-solubility, which may compromise the bioavailability and therapeutic efficacy and, thus, fail in the later stages of development [5,6]. The irregular gastrointestinal absorption of solid forms, along with the low therapeutic efficiency and possible toxicity and side-effects of polymorphs, are major concerns to overcome [7]. For instance, large differences in bioavailability among different polymorphs require different drug dosages [8]. On the other hand, the therapeutic dosage of a certain API can correspond to a toxic or potential lethal dose if the wrong polymorph is administered. Polymorphism issues result in significant economic losses in sales and in R&D to enable novel formulations back into the market [9,10].

Beyond the well-known downsides of polymorphism, the APIs’ solubility in aqueous solution, dissolution, and bioavailability are also dependent on particle size and properties [11]. Attempting to improve the drugs solubility in water as well as their bioavailability, several strategies have been investigated, especially when the oral route is envisaged [5,6]. Nevertheless, most of these strategies still use large quantities of organic solvents in the manufacturing process of these formulations, particularly to induce the crystallization of a given polymorphic form and particle size, having associated health and environmental concerns [12]. Furthermore, solvent molecules can be incorporated into the crystal structure of the API during the crystallization process [13]. Therefore, when considering the use of organic solvents, they must be removed from the API or their levels must be controlled in order to ensure human consumption safety [12]. Despite the existence of extensive literature describing novel and “greener” solvents to this purpose, there is still some reluctance by the pharmaceutical industry to accept and implement these alternatives [14,15,16].

In the above context, liquid forms of APIs are appealing solutions to avoid both polymorphism and improve low-water solubility constraints, while allowing to reduce organic solvents use. The pharmaceutical industry has relied on eutectic mixtures for this purpose, shortly exploring other options for commercialization [17,18]. In addition to these, ionic liquids (ILs) disclose high potential in the pharmaceutical field, which is mainly due to their high versatility in terms of chemical structure design towards a target application. ILs are molten salts that are composed of a large organic cation and an organic/inorganic anion. The large dimensions of their ions lead to charge dispersion, which makes difficult the formation of a regular crystalline structure [19,20]. ILs display a set of unique features, from which is possible to highlight, if properly designed, their high thermal and chemical stability and a strong solvation ability for a wide variety of compounds [21]. The proper selection of cation-anion combinations in ILs enables the use of drugs as ion components, allowing for the conversion of solid active pharmaceutical ingredients into liquid forms (API-ILs). Thus, this strategy solves the problem of polymorphism and provides improved bioavailability, and ideally boosts therapeutic properties [3,22].

**Figure 1:**
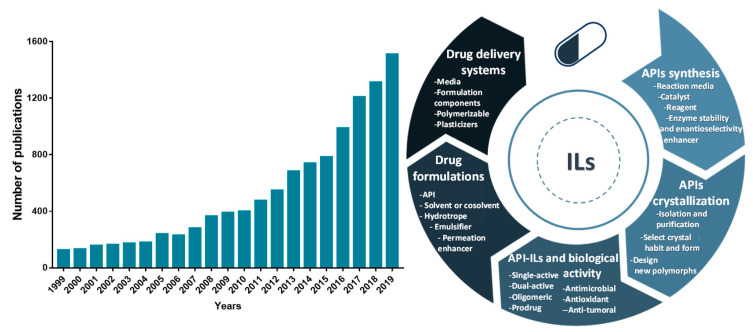
Number of publications per year in a twenty years perspective related to ILs and active pharmaceutical ingredients (APIs) (number of articles, reviews and book chapters according to a ScienceDirect database search using as keywords “ionic liquids”, “active pharmaceutical ingredients”, and “drug delivery”) (**left**). Overview of the ILs’ applications in the pharmaceutical field reported hitherto (**right**).

Because of the unique properties of ILs, their application in the pharmaceutical field has been extended far beyond the development of novel liquid forms (API-ILs), being investigated as well in other stages of drug development and delivery. The number of publications related to the application of ILs in the pharmaceutical field has grown exponentially in the past 20 years, as illustrated in Figure 1. ILs have been applied in the development of purification platforms for pharmaceuticals (an application out of the scope of this review), for which some recent review manuscripts exist [23,24,25]. Other relevant reviews and book chapters recognizing the advances of ILs in different areas of pharmaceuticals development, spanning from their formulation, biological activity, and application on drug delivery are also available [22,26,27,28,29,30,31,32,33,34]. However, most of these focus on a specific application of ILs in the pharmaceutical field. On the other hand, this review compiles and discusses the most relevant works and overall applications of ILs in the pharmaceutical field, namely on the role of ILs as solvents, reagents, and/or catalysts in the APIs’ synthesis, in the APIs crystallization, as solvents, co-solvents, and emulsifiers to improve drugs solubility, as a way of producing liquid forms of APIs, and in the development of drug delivery systems. Special attention is drawn to the most important achievements reported so far on the use of ILs in the described applications and the resulting benefits in terms of pharmaceutical formulations and pharmacological activity.

## 2. ILs in the Synthesis of Pharmaceutical Compounds

**Figure 2:**
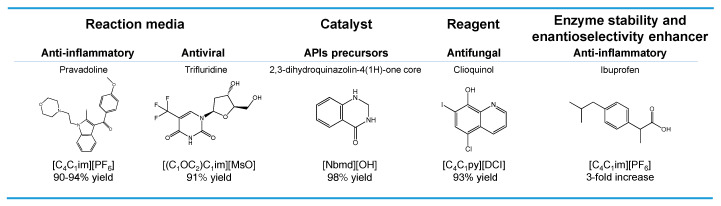
Multiple roles of ILs in the synthesis of different APIs and their respective efficiency (adequate references are given along the current section).

The increase in environmental awareness led to the proposal of the so-called Environmental factor (E-factor), which assesses the environmental impact of manufacturing processes, being defined by the ratio of the mass of waste per mass of product [35]. The pharmaceutical E-factor is one of the highest in the industry context (25–100) [35]. The waste production that is generated by the pharmaceutical industry is mainly attributed to solvent losses. In order to reduce these losses and minimize the environmental impact, it is essential to consider alternative solvents, i.e., to develop more sustainable processes. To this purpose, ILs have been studied as (i) solvents; (ii) catalysts; (iii) reagents; and, (iv) enantioselectivity enhancers in the synthesis of different APIs [36,37,38]. Reactions in these solvents may be faster and involve fewer steps than those that were carried out in conventional organic solvents, and additionally be easier to implement [39]. However, an initial assessment of conditions must be performed, since the kinetic of reactions that were carried out in ILs differ from those performed in conventional organic solvents [40]. The following described examples intend to illustrate the multifunction role displayed by ILs in APIs’ synthesis, in which some have been even combined. Figure 2 provides an illustrative summary of the applications of ILs in the synthesis of APIs and their precursors, giving one example of each application discussed in this section, with the goal of replacing the use of volatile organic solvents.

**Figure 3:**
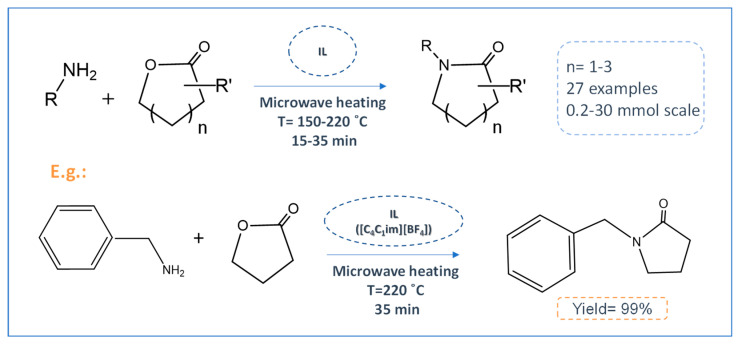
Fast and acid-free one-pot IL-based microwave methodology for direct synthesis of lactams from lactones and primary amines proposed in [41].

Given the high ILs’ applicability in different chemical processes, they have been applied in the production of pharmaceutical precursors, such as lactam [41], pyrazolone [42], thiazole [43], imidazole [44], and thiazolidine [43,45] cores, which are APIs’ precursors with vast biological activities. Due to their charged nature, ILs can provide fast microwave heating, resulting in faster and more effective reactions. For example, this approach has been successfully applied to the direct lactamization of lactones in a one-pot reaction with high yields (>80%), obtained in short reaction times (≤35 min.) (Figure 3) [41]. The combination of ILs with ultrasound irradiation can be also useful to accelerate organic reactions, allowing for synthetizing APIs’ precursors at room temperature with high yields (95%) [46].

**Figure 4:**
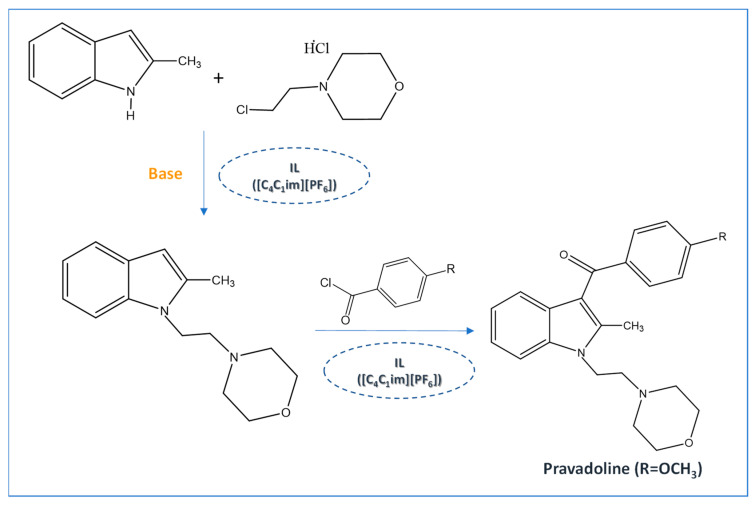
Pravadoline synthesis in an IL media proposed in [51].

The versatile nature of ILs allows for reducing the volume of solvents and the use of metal catalysts in APIs synthesis [47]. Despite the variety of cations and anions, imidazolium-based salts have been the most studied as solvents for the synthesis of APIs and their precursors [45,48,49,50,51]. In 2000, Seldon and coworkers reported the first high yield (90–94%) IL-based route to produce a non-steroidal anti-inflammatory (NSAID) drug, pravadoline, using an imidazolium-based IL as solvent, namely 1-butyl-3-methylimidazolium hexafluorophosphate ([C_4_C_1_im][PF_6_]) (Figure 4) [51]. Conventionally, the reaction to produce pravadoline is carried out in volatile organic solvents, such as dimethylformamide (DMF), while using sodium hydride as a base that additionally presents health and environmental concerns [52,53]. The proposed reaction using the IL as solvent and potassium hydroxide as base allowed for improving the conventional reaction yield (70–91%) up to 95%, simply by heating the IL at 150 °C for 2 min. With this strategy, it is possible to easily separate the API product, recycle and reuse the solvent, and the only chemical waste generated in the process is an aqueous solution of potassium chloride.

**Figure 5:**
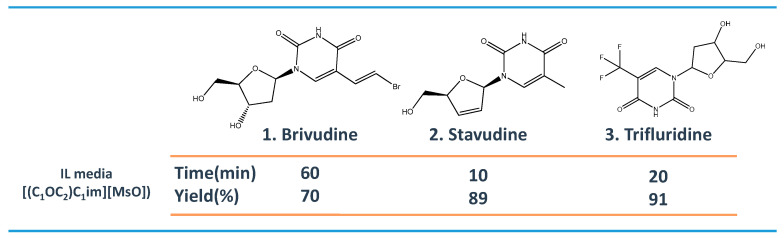
Synthesis of nucleoside-based antiviral drugs in IL media proposed in [56].

A variety of pharmaceutical agents (e.g., antibiotics, antifungals, alkaloids, or cardiac glycosides) have an heterocyclic structure to mimic the structure and, thus, the biological action of natural compounds [54]. Reactions that were carried out in IL solvent media have high regioselectivity and, for this reason, have been successfully applied in the synthesis of different heterocyclic APIs [55,56]. Imidazolium-based ILs have been used as solvents in the synthesis of antiviral drugs as brivudine, stavudine and trifluridine [56]. Figure 5 provides a summary on the synthesis time and yield of nucleoside-based antiviral drugs in IL media. Trifluridine, for example, was produced as a single product in IL media. Among others, the best results were obtained with 1-methoxyethyl-3-methylimidazolium methanesulfonate ([(C_1_OC_2_)C_1_im][MsO]), using 4-dimethylaminopyridine (DMAP) as catalyst and acetic anhydride as acylating agent. Trifluridine was obtained with 91% yield in 20–25 min. without the need of extra-purification steps. The IL was recycled and reused up to four times.

**Figure 6:**
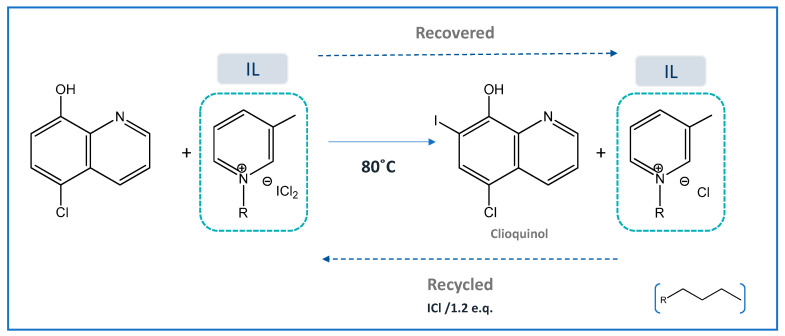
IL application as solvent and iodinating agent in the synthesis of clioquinol proposed in [57].

Antifungal and antiprotozoal drugs, such as iodoquinol and clioquinol, respectively, have been prepared by a simple and efficient iodination method, while taking advantage of the ILs’ multiple roles in synthesis, as summarized in Figure 6 [57]. The IL 1-butyl-3-methylpyridinium dichloroiodate ([C_4_C_1_py][DCI]) was used both as solvent and iodinating agent in the absence of any oxidant, catalyst, or base. It was possible to regenerate the IL for up to five runs by addition of ICl (1.2 eq.), with >90% yield, without losing its iodinating activity.

**Figure 7:**
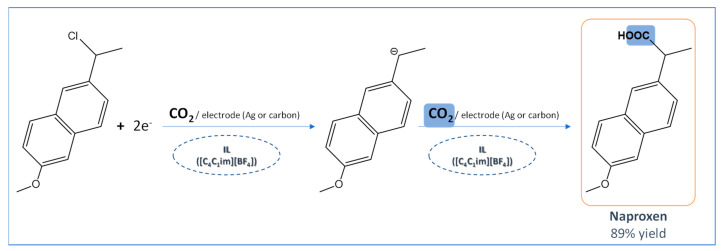
Schematic representation of naproxen’s electrosynthesis under CO2 atmosphere proposed in [60].

Naproxen was initially synthesized and commercialized by Syntex while using b-naphthol as precursor for its synthesis [58]. However, this process uses several undesirable reagents, such as nitroaromatic compounds, ammonium sulfide sodium hydride, and methyl iodide. In order to overcome these drawbacks, new procedures were considered, increasing the yields from less than 50% to 90%, but the formation of undesired side products and use of metal catalysts in these processes remained [59]. Recently, 1-butyl-3-methylimidazolium tetrafluoroborate ([C_4_C_1_im][BF_4_]) was applied as a reaction medium in the electrosynthesis of naproxen through the electrocarboxylation of 2-(1-chloroethyl)-6-methoxynaphthalene using CO_2_ [60], as summarized in Figure 7. This process allowed for achieving high yields (89%) and conversion rates (90%), with 65% of atom economy when considering the recovery of the solvent. Despite the new adapted synthesis routes mentioned above also allowing for obtaining similar high yields, the process in IL media uses cheaper and more available catalysts (electrons) and CO_2_ instead of CO, a well-known pollutant, contributing for the development of “greener” routes in the synthesis of APIs.

Biologically active compounds, with antidiuretic, anti-inflammatory, and antihypertensive activities, usually possess 2,3-dihydroquinazolin-4(1H)-one (DHQ) cores. Due to their importance for the production of such compounds, the synthesis of substituted DHQ derivatives has attracted significant attention, and different synthetic strategies have been developed. In alternative to the conventional harsh reaction conditions, gemini basic ILs (such as (4,4′-(butane-1,4-diyl)bis(4-dodecyl-morpholin-4-ium)hydroxide ([Nbmd][OH])), have been proposed as catalysts and reaction media for their synthesis [61]. In addition to the high reported yields (98%), it was shown the possibility to reuse the ILs up to five times without significant losses in catalytic activity.

Several industrial processes apply enzyme-catalyzed reactions in organic media for the production of APIs. An example of the advantageous use of ILs in biocatalysis was demonstrated in the transesterification of ribavirin, a nucleoside antiviral drug, using *C. antarctica* lipase [55]. The use of the IL [C_4_C_1_im][BF_4_] as adjuvant allowed to improve the regioselectivity, the reaction yield and reaction rate about 3.5 times. The enantioselectivity and enzyme activity in IL media was comparable or even improved when compared to the results that were obtained in traditional organic solvents. Furthermore, lipase catalyzed transterification reactions can be 25 times more effective in IL media than in conventional solvents [62].

Stereoselectivity and enantioselectivity are major concerns regarding the bioavailability and safety of pharmaceutics. In this context, ILs have been studied in order to improve the kinetic resolution of APIs and API precursors. Enantiopure chiral alcohols have been shown to be versatile chiral building blocks for the synthesis of chiral pharmaceuticals [63]. For instance, (S)-3-chloro-1-phenyl-1-propanol ((S)-CPPO) is a useful chiral building block for the synthesis of anti-depressant drugs [64]. Aiming to produce (S)-CPPO with high yields and selectivity, a variety of ILs were tested as media, where [C_4_C_1_im][NTf_2_] was ultimately selected for increasing the solubility of the (S)-CPPO’s precursor, 3-chloro-1-phenyl-1-propanone (3-CPP), in a IL/water mixture [65]. The use of the IL, allowed to dramatically increase the concentration of 3-CPP and the yield of the target compound, where the yeast reductase YOL151W was able to convert the 3-CPP enantioselectively into (S)-CPPO exclusively, with an enantiomeric excess of >99%.

The selected examples provide evidences where the use of ILs leads to comparable or superior reaction conditions and yields, and they may also simplify the separation and purification steps of some target products. The possibility to recycle and reuse ILs, without compromising the synthesis yield and lack of toxic by-products production, as shown in some examples, further reinforces the advantageous properties of ILs from the environmental and pharmaceutical perspectives. However, as happens with organic solvents, the amount of residual IL in the final product must be limited in order to guarantee the safety of the drug in the final dosage form. Following this, it is essential for a more careful monitorization of these contaminants in the final product and to study their impact in the drug’s therapeutic performance and toxicity.

## 3. ILs as Solvents, Co-Solvents or Emulsifiers for APIs Solubilization

The therapeutic efficacy of APIs is mostly defined by their solubility/bioavailability, since higher solubilities in aqueous solutions may allow for better achieving the desired concentration of a drug in systemic circulation [66]. An API is considered highly soluble when its highest dose strength is soluble in 250 mL or less of aqueous medium over a pH range 1–6.8 at human body’s temperature [67]. Nevertheless, many APIs from different pharmacological classes do not fulfill these requirements and they are only sparingly soluble in water. Although water is the preferable medium when considering human consumption, occasionally the use of non-aqueous solvents is considered since their presence may positively influence the API absorption [68]. Organic solvents like ethanol, methanol or dimethyl sulfoxide (DMSO) are generally used as solvents or co-solvents in pharmaceutical formulations to improve solubility [30]. To address this solubility challenge, ILs have been investigated as alternative neat solvents [69]. The good solvation ability of ILs also allowed for increasing the aqueous solubility of APIs by cosolvency, hydrotropy and micellization phenomena, as summarized in Table 1. ILs represent a novel class of hydrotropes with superior performance to enhance the solubility of poorly water-soluble compounds in aqueous solution, driven by the formation of API–IL aggregates [21]. Cosolvency, unlike the hydrotropic mechanism, is not based on the formation of aggregates, but on the solvation of the solute by a mixed solvent (water + IL), acting by disrupting the water self-association and by reducing the interfacial tension between the API and the solvent medium [70]. The use of surface-active agents, on the other hand, acts by taking advantage of their amphiphilic nature and by incorporating hydrophobic APIs into the micelles core [71]. It has been demonstrated that the selection of IL anion/cation combinations has a significant impact on the solubilization mechanism of poorly water-soluble APIs and solvation ability, as shown by solubility enhancements for antifungal, analgesic, and nonsteroidal anti-inflammatory drugs that are listed in Table 1. In particular, it has been demonstrated that the solubility of drugs like amphotericin B, albendazole, itraconazole, paclitaxel, or etodolac, which are very low-water soluble, can be enhanced by several orders of magnitude (from 700–5.6 × 10^6^-fold) by adding ILs.

The application of pure ILs for APIs solubilization was first reported in 2008 by Jaitely et al. [85], who investigated the ILs [C_4−8_C_1_im][PF_6_] on the solubilization of potassium penicillin V, dexamethasone dehydroepiandrosterone, and progesterone. Although these ILs are immiscible in water, it is possible to use these formulations to enhance the release of some solutes into an aqueous medium. The studied ILs were submitted to a current flow (over the range 1–5 mA) allowing to increase the release rate of the APIs from the IL to the aqueous medium. The release of these APIs slowly increased with the increase of the alkyl chain length of the IL cation (up to three-fold). The partition of APIs in the studied ILs/water systems was further correlated with the APIs octanol-water partition coefficient. To infer the safety of these solvents for pharmaceutical applications, their cytotoxicity was evaluated in Caco-2 cell lines, which suggests that, with the exception of 1-octyl-3-methylimidazolium hexafluorophosphate ([C_8_C_1_im][PF_6_]), these compounds are non-toxic (90% cell viability) at the conditions and concentrations studied, and might be considered as excipients in pharmaceutical formulations.

The same trend was observed by Mizuuchi et al. [69], who studied similar ILs in order to solubilize albendazole, danazol, acetaminophen, and caffeine. The increase in the hydrophobicity of the imidazolium cation ([C_4−8_C_1_im]^+^) resulted in a higher solvation ability for hydrophobic drugs. However, this trend resulted in a decrease in the solubility of hydrophilic drugs. The authors demonstrated that it is possible to increase albendazole’s solubility more than 37,000-fold while using [C_8_C_1_im][PF_6_] as solvent. In a different study by Forte et al. [82], the variation of the anion in 1-decyl-3-methylimidazolium-based ILs and the alkyl chain length of the cation ([C_2-10_C_1_im]^+^) were studied to infer their effect on isoniazid’s (antibiotic) solubility. The results showed that the presence of an acidic proton at the 2-position of [C_2_C_1_im]^+^ increases the ILs ability to hydrogen-bond with isoniazid, leading to higher solubility values. Among the ILs studied, 1-decyl-3-methylimidazolium trifluoromethanesulfonate ([C_10_C_1_im][CF_3_O_3_S]) was found to be the best solvent for isoniazid (at T > 38 °C). Furthermore, the increase of the alkyl chain length at the imidazolium cation decreases the acidity of the proton at the 2-position, thus increasing the API’s solubility in the IL. Overall, the trends that were obtained in the described studies demonstrate that the influence of the cation’s alkyl chain length differs according to the IL and the APIs nature, and accordingly with the molecular-level mechanisms involved. These differences make difficult the establishment of heuristic rules and development of predictive models that could be used in a widespread manner.

Although interactions between the IL anion and the API have been reported as relevant, mainly via hydrogen-bonding, the influence of the IL anion in which the IL hydrogen-bond basicity can be strongly tuned is less studied and still not completely understood. Although promising results have been reported in terms of APIs solubility, comparisons between the results obtained in IL media with the ones that were obtained with other common solvents, such as water or ethanol, are shortly explored along with the bioavailability profiles. These assays should be considered in order to support the advantageous use of IL-based solvents alternatives.

In addition to the study of pure ILs as solvents for APIs, the application of ILs as co-solvents has been investigated. For instance, cholinium-based ILs have been successfully applied as co-solvents in paclitaxel formulations for chemotherapeutic treatment [83]. Paclitaxel is a low-water soluble API (<4 μg mL^−1^) that is used in the treatment of different types of cancer; however, its parenteral formulation requires the use of ethoxylated castor oil (CrEL) and ethanol as solubilizing agents. The use of these agents can be associated with major hypersensitivity reactions and side effects. By using cholinium-amino-acid-based ILs as co-solvents, it was possible to remarkably enhance the APIs solubility in aqueous media (>5500-fold) and decrease the formulation toxicity and hypersensitivity, while maintaining the antitumor therapeutic action of the API. In a different study, also using amino-acid-based ILs, and in particular cholinium tryptophan, it increased the glibenclamide’s (an antidiabetic drug) solubility in aqueous solutions with 6.5 wt% of IL from 400 to 2000-fold [80]. The establishment of hydrogen bonds and π–π interactions between the API and the IL anion were described as playing a major role in the obtained solubility improvements in both studies.

In a more fundamental study, tetrabutylammonium-, phosphonium-, imidazolium-, pyridinium-, piperidinium- and pyrrolidinium, and cholinium-based ILs were investigated in aqueous solution regarding their ability to act as hydrotropes and improve the solubility of ibuprofen [86]. It was found that the IL cation and anion synergistically contribute to the hydrotropic mechanism of solubilization. Among the cations that were investigated in a chloride-based IL series, imidazolium- and phosphonium-based ILs lead to a higher increase in the drug solubility. In order to evaluate the influence of the IL anion on ibuprofen’s solubility, [C_4_C_1_im]- and sodium-based hydrotropes were investigated, where a higher hydrotropic activity was disclosed with 1-butyl-3-methylimidazolium thiocyanate ([C_4_C_1_im][SCN]) and 1-butyl-3-methylimidazolium dicyanamide ([C_4_C_1_im][N(CN)_2_]). These ILs increase the aqueous solubility of ibuprofen by 60- and 120-fold, respectively, in comparison to the API in pure water. The formation of IL-drug aggregates was proved based on dynamic light scattering and molecular dynamics simulations. In particular, ILs have been reported as powerful catanionic hydrotropes [23,86].

The use of surfactants above the critical micelle concentration (CMC) can be also an appealing alternative to increase API’s solubility. By varying the cation type and alkyl chain length and the nature and size of the counterion it is possible to change the ILs’ hydrophilic-hydrophobic balance. ILs with surfactant behavior are usually referred to as surface-active ionic liquids (SAILs), displaying high potential to increase the solubility of pharmaceutical agents in aqueous media. Sanan et al. [87] studied 1-dodecyl-3-methylimidazolium chloride ([C_12_C_1_im]Cl)-ibuprofen mixtures in aqueous solution, ranging from monomeric to micellar regions. Aggregate assemblies in aqueous media (mixed micelles) were observed, depending on the mixture composition. The formation of these complexes was mainly attributed to the establishment of hydrophobic, electrostatic and hydrogen-bonding interactions. Faria et al. [88] investigated surface-active ILs, both cationic and anionic, as well as composed of different cations and anions, for the solubilization of the nutraceutic ursolic acid in aqueous media. For this, different ILs constituted by long alkyl side chains with known surface-active characteristics ([C_8-18_C_1_im]X with X = Cl and [C_8_H_17_SO_4_]) and tributyltetradecylphosphonium chloride ([P_444(14)_]Cl) were considered. The use of the best SAIL aqueous solutions allows for solubility enhancements of ursolic acid in 8 orders of magnitude when compared to pure water. More recently, aqueous solutions of [C_12_C_1_im]Cl were used to increase the solubility of the nutraceutical oleanolic acid [89]. An increase in the IL concentration up to 1000 mM improved the solubility of oleanolic acid to 21.10 mg mL^−1^, indicating that aqueous solutions of SAILs leads to a remarkable increase (up to 10^6^-fold) on the solubility of the target compound in water.

Although significant results have been disclosed on the use of ILs as solvents, co-solvents, or surfactants to improve the APIs solubility, most studies reported so far focus on imidazolium-based ILs. This trend is probably associated with the fact that these ILs are commercially available, and well studied and characterized in literature. Although few other IL combinations were investigated, the results reported hitherto on drug solubility enhancements promote the evolution of ILs further from their solvent applications to the study of novel drug delivery approaches, where stability, absorption, and bioavailability can be improved. Furthermore, special care must be taken for the mutual administration of ILs and APIs, since these can also induce multixenobiotic/multidrug cell resistance and/or reduce the effectiveness of therapies, an issue that is usually not addressed in the reported studies. More biocompatible combinations are expected to be studied and more complete studies are still required to boost IL research and enable their use in pharmaceutical formulations.

## 4. ILs in APIs Crystallization

**Figure 8:**
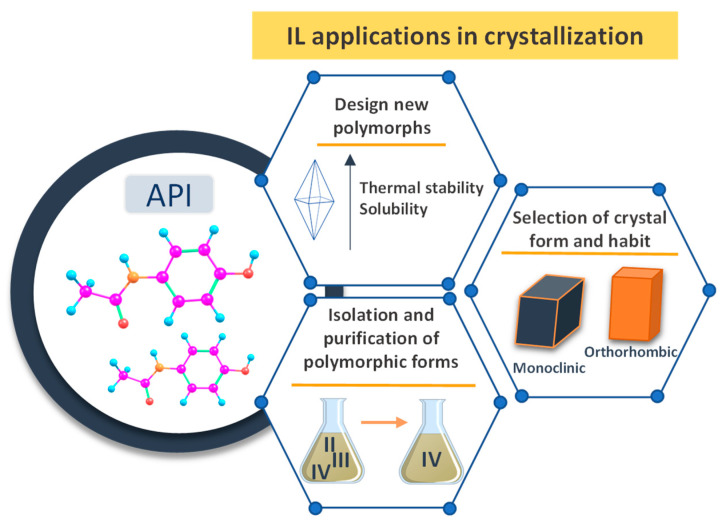
Applications of ILs in APIs’ crystallization processes (adequate references are given along the current section).

The properties of crystalline APIs, such as their solubility, structural stability, and dissolution rates, are mainly dependent on the respective polymorphs [8,90]. Thus, designing the correct polymorph represents a critical requirement in drug development and production as it impacts the APIs bioavailability and shelf life. Crystallization from organic or aqueous-based reaction media is often a key step in pharmaceuticals isolation and purification [91]. APIs polymorphs can be originated from the variety of intermolecular interactions between the molecules, which are dependent on the crystallization conditions (solvent, temperature, additives, and supersaturation) [92]. It is possible to fine-tune the polymorphic form by adjusting the crystallization conditions, introducing guest molecules or promoting a preferred crystal nucleation while using additives [93]. Several studies have reported the application of ILs as adjuvants in APIs crystallization [94,95,96], allowing for not only the design of new polymorphic forms, but also to manipulate the crystal form and habit to present enhanced properties, and ultimately, to separate and isolate specific polymorphic forms that are not achievable with conventional solvents (Figure 8). IL-based crystallization techniques, which include solvent-antisolvent [97], cooling crystallization [98], or drowning-out [92] techniques, were proposed in order to promote the correct habit and polymorphic forms of several drugs.

The use of ILs has shown the possibility to design new APIs’ polymorphs with enhanced thermal stability [92]. The IL 1-allyl-3-ethylimidazolium tetrafluoroborate ([(CH_2_CH=C_2_)C_2_im][BF_4_]) has been applied to design polymorphs of adefovir dipivoxil, through drowning-out crystallization. This process can be considered to be one of the most important techniques to be applied when the separation of solutes from multicomponent solutions is envisaged [99]. This method relies on the supersaturation of the solution by adding specific substances, drowning-out agents to the initial solution in order to reduce the solubility of the solute. The use of the IL in the crystallization process allowed for obtaining the form-II of the API, and upon an increase of the crystallization temperature two new polymorphic structures and hydrated crystals form. A significant increase in the thermostability in aqueous solutions was verified when using the IL.

By the application of the IL [(CH_2_CH=C_2_)C_2_m][BF_4_] as solvent and 1-butyl-2,3-dimethylimidazolium tetrafluoroborate ([(C_4_C_1_C_1_m][BF_4_]) as antisolvent, a new form of adefovir dipivoxil crystal was obtained at a crystallization temperature below 50 °C [97]. This new polymorphic form was achieved due to unique intermolecular interactions between API molecules that were promoted by the IL, resulting in different molecular packing during crystallization. However, when considering the use of the ILs [(CH_2_CH=C_2_)C_2_m][BF_4_], 1,3-diallylimidazolium tetrafluoroborate ([(CH_2_CH=C_2_)_2_im][BF_4_]) and 1-ethyl-3-methylimidazolium ethylsulfate ([C_2_C_1_im][EtSO_4_]), the conventional form-I polymorph was obtained. The possibility to use high crystallization temperatures enabled the formation of a stable polymorph, with enhanced solubility and thermal stability over 100 °C (two-fold increase in the decomposition temperature). Using a similar approach, rifampicin nanosized particles were prepared to improve the solubility and dissolution kinetics of poorly-water soluble APIs [100]. To this purpose, the use of the IL 1-ethyl-3-methylimidazolium methylphosphonate ([C_2_C_1_im][CH_3_OHPO_2_]) as alternative solvent and phosphate buffer as an antisolvent was considered, allowing for obtaining API crystals with <1 µm. Reducing the particle size down to the submicron range allowed not only a faster dissolution ability, but also to increase the APIs’ solubility by 30%.

Imidazolium-based ILs have been studied to control the crystallization of gabapentin, a neuroleptic drug used to treat epilepsy [101]. This API presents three polymorphic forms (forms II, III, and IV) and a hydrated form (form I) [102]. The commercialized polymorph is the form II due to its highest thermodynamic stability [103]. However, forms III and IV are commonly obtained through crystallization in ethanol at room or high temperature, where the form IV cannot be completely isolated [102]. Distinct IL cation/anion combinations were studied in order to access their ability in directing the crystallization process towards the isolation and stabilization of less stable polymorphs [101]. Using 1-hexyl-3-methylimidazolium tetrafluoroborate ([C_6_C_1_im][BF_4_]), the API form IV was isolated, which is a highly unstable polymorph.

ILs have been investigated as well for the isolation of specific polymorphic forms of paracetamol [94]. Imidazolium-based ILs were studied regarding their impact on the API’s solubility, mainly by tailoring their hydrogen-bond ability with the API. Because the solubility of the API was shown to be governed by the basicity of the IL anion, 1-ethyl-3-methylimidazolium acetate ([C_2_C_1_im][CH_3_COO]) was then selected for the crystallization process. The strong interactions between the IL and the studied antisolvents (ethanol, acetic acid, and 1,1,1,3,3,3-hexafluoroisopropanol) allowed for engineering the crystallization in the form of polymorph I (the most stable) and manipulating the system’s interactions to obtain crystallization yields greater than 88% at room temperature.

Attempting to control the crystallization of paracetamol, Smith et al. [104] studied the ILs [C_4_C_1_im][PF_6_] and 1-hexyl-3-methylimidazolium hexafluorophosphate ([C_6_C_1_im][PF_6_]). The selected ILs, the respective concentration, and the method of crystal growth considered (cooling crystallization) have shown impact on the crystal habit and size. When [C_6_C_1_im][PF_6_] was used at the lowest concentration (16 mg mL^−1^), tetragonal bypyramids were formed; by increasing the concentration of the IL, it was possible to move from plate particles to more tubular structures (30–69 mg mL^−1^) as a consequence of growth post spontaneous nucleation. At higher concentrations, ≈ 69 mg mL^−1^, particles with 23 to 206 µm were obtained.

To avoid the use of an antisolvent, Weber et al. [98] proposed the cooling crystallization in IL media, with 1-ethyl-3-methylimidazolium bis(trifluoromethanesulfonyl)imide ([C_2_C_1_im][NTf_2_]), also acting as a purification methodology for twelve APIs with anti-inflammatory, antifungal and antipyretic properties. From those, ten of the APIs proved to be highly soluble in the IL at their melting temperature. Among the studied systems, similar or higher purities with improved yields to those that were obtained with antisolvent crystallization were achieved, enabling the possibility to use this method for APIs purification.

Despite the promising results reported so far and the growing interest on the use of ILs to tailor the APIs polymorphs, there is still a gap in a systematic and comprehensive research on this topic, particularly to better understand the IL cation and anion effects. The use of computational methods can be an advantageous alternative to understanding the IL-API interactions driving the formation of specific polymorphic forms and habits, as it is already performed for other conventional solvents [105]. Furthermore, the development of effective separation methods and research on techniques to avoid the IL contamination in the final product are highly demanding.

## 5. ILs with Biological Activity

**Figure 9:**
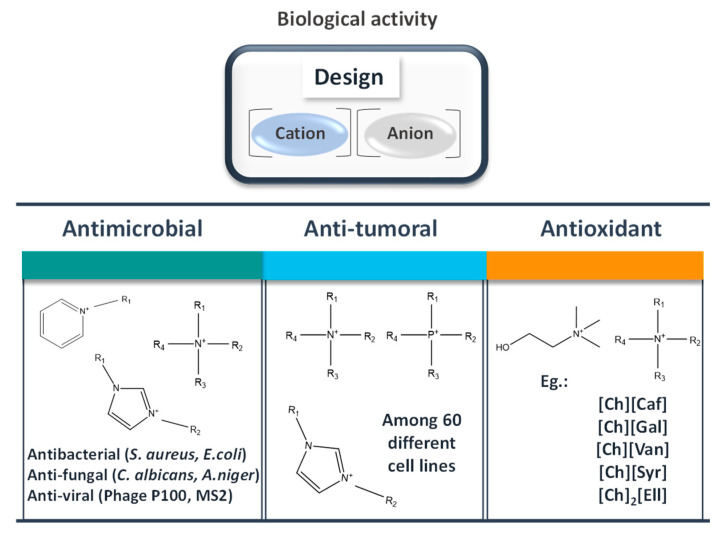
Biological activities reported for ILs and the respective studied cations (adequate references are given along the current section).

Because of the myriad of anion-cation combinations in ILs, these solvents may display specific biological activities [106]. Although the application of ILs in this area is emerging, promising results were already disclosed. Figure 9 provides an overview on the biological activities of ILs, namely antioxidant, anti-tumoral, and antimicrobial activity, covering some of the main types of cation-based ILs studied so far.

Imidazolium- and pyridinium-based ILs have been largely investigated in what regards their antibacterial activity [107,108,109,110]. Although some exceptions may appear and have been reported, in general, an increase in the alkyl chain length of the IL cation (1-alkyl-3-methylimidazolium based ILs ([C_4-8_C_1_im]^+^) and 1-alkyl-N-methylpyrrolidinium based ILs ([C_4-8_C_1_pyr]^+^)) leads to higher antibacterial activity. Furthermore, ILs cations with longer aliphatic chains and more alkyl group substituents on the cation ring exhibit higher antibacterial activity against Gram-positive bacteria (e.g: *Staphylococcus aureus*, *Bacillus subtilis*) and Gram-negative bacteria (e.g.,; *Escherichia coli*, *Pseudomonas fluorescens*) and *Saccharomyces cerevisiae* [110]. From these, *B. subtilis* demonstrated the higher susceptibility to the tested ILs. Later studies with ILs with longer alkyl chain lengths (1-alkyl-3-methylimidazolium based ILs ([C_8-14_C_1_im]^+^) and 1-alkyl-N-methylpyrrolidinium based ILs [C_8-14_C_1_pyr]^+^)) provided further insights on the antimicrobial activity of ILs [111]. Overall, the effect of the tested ILs against Gram-positive microorganisms was similar or even higher than that displayed by a common antimicrobial agent, cetyltrimethylammonium chloride. Based on the exposed, the ILs antibacterial efficiency can be tuned by varying both the alkyl chain length and modifying the head group at the cation. The increase in the susceptibility of these pathogens can be assigned to the ability of ILs to interact or disturb biological membranes, which leads to cell death [112]. In all of these cases, varying the anion identity did not reveal a significant effect on the ILs’ antimicrobial activity.

Doria et al. [113] synthesized a series of *N*-cinnamylimidazolium salts with different alkyl chain lenghts (1, 6, 8, and 10 carbons), and evaluated their antibacterial activity against skin and soft tissue infections. These ILs were synthesized while employing microwave radiation under solvent-free conditions, attempting to minimize the environmental concerns that were related to conventional IL synthesis. Based on antimicrobial activity studies, it was possible to verify that as the alkyl chain is increased, the antibacterial activity increased with a dose-dependent effect. Molecular dynamics simulation studies allowed for explaining this antimicrobial behavior, as the result of the ILs’ insertion in the lipidic double layer, facilitating the subsequent diffusion to the intracellular space. While higher aliphatic chain lenght ILs extend to the interior of the membrane, the lack of hydrophobicity of lower alkyl chain lenght ILs reduces this phenomenon [113].

More recently, predictive models for the antimicrobial and antifungal activities of ILs started to be developed, aiming at a rational design of ILs to be included in pharmaceutical applications. Cho et al. [114] reported six quantitative structure-activity relationship (QSAR) models, which were developed while using linear free energy relationship (LFER) descriptors calculated by density functional theory and a conductor screening model, to predict the minimal inhibitory concentration (MIC) and minimal biocidal concentration (MBC) of ILs against *E. coli* and *S. aureus.* Later, QSAR and molecular docking were applied in order to address the antibacterial activity of 131 imidazolium-based ILs against *S. aureus* ATCC 25923 and its clinical isolate [115]. The developed models presented robustness, predictive power and reliability, with 80–82% of accuracy. The obtained results reveal that ILs with C_12_ alkyl chains or with two identical C_8_ and C_9_ alkyl chains seem to have higher activity against the pathogen, and they can be foreseen to design novel strategies against this microorganism. Attempting to investigate IL candidates as antibacterial agents, Zheng et al. [116] studied a series of ILs by molecular dynamics. The cations of ILs were found to insert into the lipid bilayer spontaneously, regardless of the cation types. Furthermore, imidazolium-based ILs with different alkyl chain lengths not always keep the preferential orientation, presenting the alkyl side chain of the cations close to the tail groups of the bilayer and the imidazolium ring close to the head groups of the lipid bilayer. This spontaneous insertion and reorientation inside the lipidic bilayers might be the cause of disorder and disruption of membranes and, thus, influence antibacterial activity [116].

In addition to antibacterial properties, ILs were disclosed as presenting antiviral activity. A systematic analysis investigated the effects of defined structural elements of 55 ILs (by changing the cation core, anion, and the length of the cation alkyl side chains) on virus activity, namely on the human norovirus surrogate phage MS2 and phage P100, representing non-enveloped DNA viruses [117]. Imidazolium-based ILs ([C_1-10_C_1_im]Cl) did not show particular effectiveness against the phages, except for the IL with a higher alkyl chain cation that exhibited a reduction in the phages number. The antiviral activity shown to be IL concentration-dependent. Because the phages used in the previous study are nonenveloped, the observed inactivation by long alkyl chain ILs could be mainly attributed to protein denaturation (as the capsid of the phages consists of proteins), rather than membrane disturbance typically caused by surfactant-like behavior. However, the effect of the IL anion on the antiviral activity remains unclear.

Despite their antibacterial and antiviral properties, some ILs can present antifungal activity, even at low concentrations (0.28 µg mL^−1^). Bergamo et al. [118] reported an in vitro antifungal activity of 1-hexadecyl-3-methylimidazolium chloride ([C_16_C_1_im]Cl) against multidrug-resistant *Candida tropicalis* isolates, whereas other authors [107] have shown the potential of [C_6-14_C_1_m]Cl ILs to control planktonic bacteria and biofilm formation. ILs with tetraalkylammonium and pyridinium cations were combined with anions that were derived from artificial sweeteners (saccharinate and acesulfamate) attempting to pair the biological activity inherent in the cation with the anion’s biological function [119]. These ILs were tested for their antifungal activity against *C. albicans*. However, these ILs present equal or decreased antifungal activity towards the microorganism than the starting compounds. Previous findings have shown that the IL effect on fungal metabolism is more intricated than the attribution of their activity to the ILs’ toxicity [120]. In this regard, Suchodolski et al. [121] synthesized novel menthol-based ammonium ILs ([C_10-12_-Am-Men]Cl) attempting to understand the mechanisms underlying their antifungal activity on *C. albicans*. As it happens for the previous mentioned works, the antimicrobial activity increased with the increase of the alkyl chain length of the cation, being the most effective ILs the ones that present more than 11 carbon atoms. When used at 50 µM, these ILs cause partial decomposition of the cell wall and promote the detachment of fungal cells. These novel ILs can be considered as disinfectants due to their antifungal activity and low hemolytic activity.

Antioxidant properties have gained interest in the pharmacological context due to the possibility to reduce the free radicals’ concentration at skin, and thereby preventing and repairing damages caused by oxidative stress. Some phenolic compounds present high antioxidant and anti-inflammatory activities; however, their limited aqueous solubility represents a disadvantage towards their incorporation in water-rich pharmaceutical formulations and skin care products. To overcome these drawbacks, phenolic acids have been considered as IL anion sources to increase their solubility and antioxidant activity. However, it should be remarked that due to their action and when considered for therapeutic purposes, these may be also considered as new active principle ingredients in the form of ILs (API-ILs), which are discussed below. Overall, ILs have proven to possess high antioxidant activities, as summarized in Table 2. Sintra et al. [122] synthetized five cholinium-based ILs using gallate, caffeate, vanillate, syringate, and ellagate anions. The resulting ILs demonstrated a solubility in water ≈ 3 orders of magnitude higher than the corresponding phenolic acids. These ILs presented not only similar, but even higher antioxidant activities, as well as comparable cytotoxicity and lower ecotoxicity profiles than their acidic precursors, being the most promising results obtained with the IL dicholinium ellagate. In another study, hydroxyl functionalized ammonium dicationic ILs containing natural derived ions and ether linkage between cationic head groups were synthesized and described as a novel form of antioxidants, where protocatechuic acid (also known as 3,4-dihydroxybenzoic acid), a natural compound with not only antioxidant properties but also chelation ability, was considered as anion [123]. All of the in vitro studies indicated that the antioxidant activity of dicationic ILs was significantly higher than that of free acids or commonly used antioxidants. More recently, attempting to understand the effect of the cation structure towards the antioxidant activity, Ahmad et al. [124] evaluated five ferulate-based ILs with ammonium cations comprising different alkyl chain lengths. The prediction of their antioxidant activity based on the σ-potential of the COSMO-RS model was in agreement with the experimental DPPH free radical scavenging results, revealing that tertiary alkanolamine-based ILs have higher antioxidant activities than secondary alkanolamine-based ILs. All of the synthesized ILs showed higher antioxidant activities than the ferulic acid precursor, even at low concentrations (up to 12.93 µM). In a different approach, novel ILs derived from natural sources were designed to enhance other biological properties in aqueous solutions, such as analogues of glycine-betaine (AGB-ILs) [125]. AGB-ILs, namely triethyl [2 -ethoxy-2-oxoethyl]ammonium bromide ([(C₂)₃NC₂]Br), have recently been studied regarding their potential to enhance anti-inflammatory and antioxidant activities. These ILs allowed for increasing the antioxidant/anti-inflammatory activities of nutraceutical extracts, being possible to use them in nutraceutical formulations.

ILs have been largely tested in different cell lines in order to assess their antitumoral activity. From these, cancer cells lines have provided relevant results towards the understanding of the ILs potential as anticancer agents [126]. Phosphonium-, tetralkylammonium-, and, later, imidazolium-based ILs have been tested in vitro in 60 human tumor cell lines [126,127]. Phosphonium-based ILs were found to have higher anti-tumor activity than ammonium-based ones. Imidazolium-based ILs showed particularly high activity against leukemia cell lines, whereas an increase in the alkyl chain length leads to significant improvements in antitumor activity. Thus, multiple possibilities of IL cation-anion combinations can rule the resulting biological activity and associated cytotoxicity, playing a major role in therapeutic applications. The apoptotic mechanism that was caused by alkyl-methylimidazolium-based ILs was later unveiled using rat pheochromocytoma (PC12) cells [128].

As the analysis of the biological activity of ILs is strongly dependent on the organism considered, it is important to consider a broader study for each IL in order to obtain a complete profile of its activity. The proper cation/anion combination will allow for the synthesis of ILs with specific pharmacodynamic properties. These ILs with biological properties can be applied not only as novel active compounds, but ideally, and by taking advantage of their solubilization ability, be designed to enhance the solubility of a given API and supplement and/or potentiate their therapeutic action. Furthermore, a closer look is required towards the understanding of ILs’ distinct mechanisms of membrane binding, insertion, and disruption.

## 6. API-ILs as Liquid Forms of APIs

**Figure 10:**
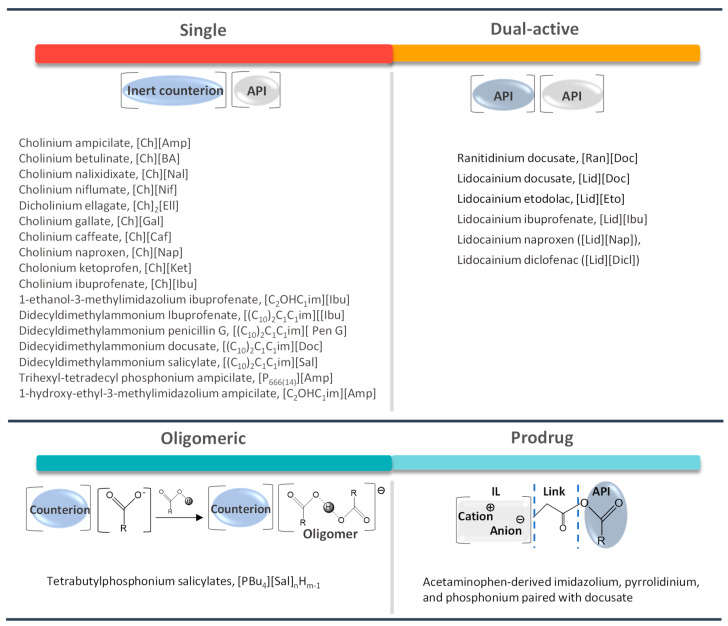
Schematic examples of API-ILs pharmaceutical formulations (adequate references are given along the current section).

The design of novel liquid forms of APIs where they are at least one the constituting ions in ILs (API-ILs) is an appealing strategy to overcome the solubility, bioavailability, and polymorphism drawbacks. Their charged and liquid state allow to overcome the melting enthalpy barrier and improve solubility/bioavailability. The large variety of IL cation–anion combinations allows for obtaining new drugs in the form of API-ILs with specific physicochemical and biological properties, and even to possess dual pharmacological action. These ILs can be also obtained using oligomeric ions or by applying the prodrug strategy to one of the ions of an API-IL. Figure 10 summarizes the options of design for API-ILs found in the literature. During this section, several alike summarizing images will be provided with the information collected from the works to be discussed.

API-ILs were first reported by Rogers and coworkers [129] in 2007, with the synthesis of ranitidine docusate ([Ran][Doc]), liquid at room temperature. Ranitidine, which is a histamine H_2_-receptor antagonist, is well known for its polymorphic conversion that affects its pharmaceutical action. The authors demonstrated its conversion into an IL form by incorporating the docusate anion, which, in addition to overcoming the polymorphism concern, also improves the API absorption. After this pioneering work, an increasing number of reports on API-ILs emerged over the years, where the structural and chemical properties of the ions and a wise selection of counterions led to the formation of IL forms of the pristine drugs, with single or dual therapeutic action [130,131,132,133,134]. However, it should be taken into account that, upon dissolution, the combination of APIs with simple and inert counterions or other active agents will dissociate in the body fluids, and the cationic and anionic components will follow their independent pharmacokinetic and metabolic pathways [135].

**Figure 11:**
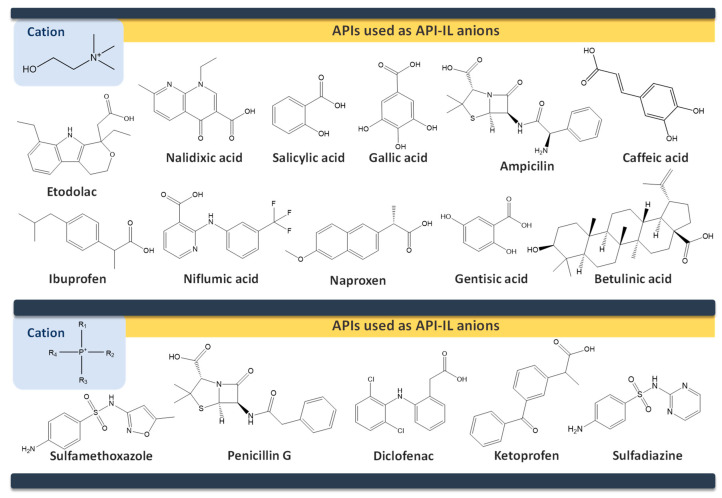
Selected examples of cations and anions used in API-ILs preparation (adequate references are given along the current section).

API-ILs of different pharmacological classes have been reported, including the following APIs: lidocaine [106,136], sulfacetamide [129,137], ibuprofen [137,138], indomethacin [139], procaine [136], aspirin [131,136], salicylic acid [136], piperacillin [137], penicillin G [137], and docusate [138]. Most of the reported works comprise the API as the anion combined with an “inert” cation, as summarized in Figure 11, such as cholinium or phosphonium cations. These cations are mostly explored since they are widely characterized in the literature and, especially, in the case of cholinium, this is a safe and low-cost cation source. However, the possibility to predict if the selected ions will result in an API-IL and their resulting properties is still a challenge to be tackled.

**Figure 12:**
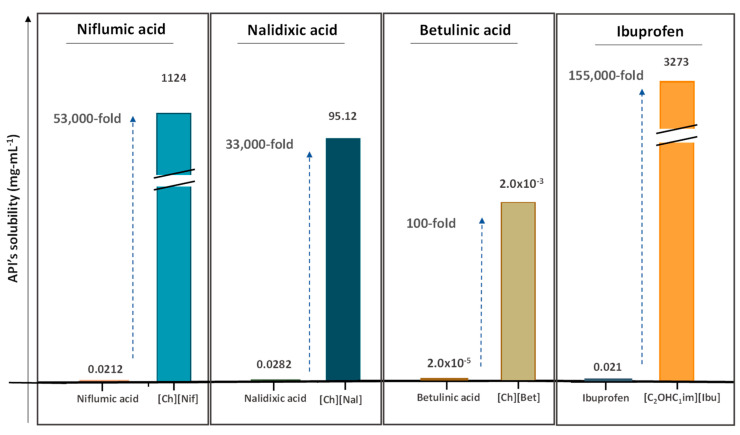
Example of solubility enhancements provided by API-ILs in aqueous media in comparison with the parent APIs and their dissolution in water (mg mL^−1^) (adequate references are given along the current section).

In general, API-ILs exhibit improved solubility in water and bioavailability when compared with the original and non-charged APIs. Figure 12 depicts examples of promising solubility enhancements that are achieved by this approach, which can be advantageous alternatives for different pharmacological classes. As an example, betulinic acid, a low-water soluble natural product with anti-cancer, anti-inflammatory, and anti-HIV properties, has been converted into an API-IL using cholinium as counterion [140]. The cholinium-based derivative has a significantly higher solubility in water than betulinic acid (by 100-fold) and its half maximal inhibitory concentration (IC_50_) was considerably improved (from 60 to 22 μg mL^−1^), meaning that a higher ability of liberating its latent biological activity for inhibition of HIV-1 protease. The API-IL approach has been also applied to increase the solubility of nalidixic acid by its conversion into cholinium nalixidixate ([Ch][Nal]) and of niflumic acid by its conversion into cholinium niflumate ([Ch][Nif]), where an increase of 3300-fold and 53,0000-fold in solubility in aqueous media, respectively, was observed [141]. Furthermore, the in vitro study on two human cell lines, Caco-2 colon carcinoma cells and HepG_2_ hepatocellular carcinoma cells, revealed that the cytotoxicity of these APIs is preserved upon their conversion into ILs. Other poorly water-soluble APIs, such as diclofenac, ibuprofen, ketoprofen, naproxen, sulfadiazine, sulfamethoxazole, and tolbutamide, were converted into tetrabutylphosphonium-based ILs, and their solubility in water as compared to the free acids and sodium salts [142]. Tetrabutylphosphonium-based ILs improve the solubility of the corresponding API in aqueous media, where significantly higher maximum concentrations were reached with ibuprofen (≥80-fold), ketoprofen (≥60-fold), naproxen (≥70-fold), sulfadiazine (130-fold), and sulfamethoxazole (≥50 fold). In another study, the conversion of ibuprofen into the IL 1-ethanol-3-methylimidazolium ibuprofenate ([C_2_OHC_1_im][Ibu]) allowed for increasing the API’s solubility in water by 155,000-fold in comparison to the original API [143].

Ferraz et al. [144] reported the conversion of ampicillin into ampicillin salts/ILs, including API-ILs liquid below body’s temperature, namely trihexyltetradecylphosphonium ampicillin ([P_666(14)_][Amp]) and 1-hydroxy-ethyl-3-methylimidazolium ampicillin ([C_2_OHC_1_im][Amp]), as well as a water-soluble alternative with low melting temperature (T_m_ = 58 °C), cholinium ampicillin ([Ch][Amp]). Later, the water solubility (at room and body’s temperature) and the respective hydrophilic/lipophilic balance of these API-ILs was evaluated [145]. The solubility for [Ch][Amp] and [C_2_OHC_1_im][Amp] was found to be comparable to the water solubility of the respective ampicillin sodium salts and it increased by nine-fold at body’s temperature in comparison with the pure salt, thus standing as a competitive alternative to the marketed drug. For both ILs, an enhancement in the octanol–water partition coefficient by 11- and 7-fold was relatively verified to the starting ampicillin. The authors also evaluated the antibacterial activity of the prepared ampicillin-based ILs [146]. The studied ILs showed increased growth inhibition for some Gram-negative resistant bacteria, achieving MIC values 10–1000 higher than the ampicillin salt, including the action against resistant bacterial strains. More recently, the anti-tumoral activity of these ampicillin-based-ILs was assessed in human cancer cell lines in order to evaluate the possibility to have multiple and enhanced biological activity in the liquid form. To this purpose, the anti-tumoral activity was assessed in T47D (breast), PC3 (prostate), HepG2 (liver), MG63 (osteosarcoma), and RKO (colon) cell lines [147]. [C_2_OHC_1_im][Amp] shown to be the most relevant ampicillin-based IL, with a higher antiproliferative activity and lower cytotoxicity being associated towards healthy cells.

Following the previous reports with ampicillin [144,145,146,147], primaquine-based ILs were synthesized aiming to treat malaria infection [148]. Primaquine blocks disease transmission due to its gametocytocidal action; however, its action is overshadowed by toxicity issues and by the drug’s poor activity against blood-stage parasites. Aiming to tackle these drawbacks, the API was combined with different cinnamates as counterions and screened against blood-stage chloroquine-sensitive and chloroquine-resistant *Plasmodium falciparum* parasites. The novel API-ILs showed an increased in vitro blood-stage activity when compared to the pure API. Later, it was discovered that primaquine-derived ILs may contribute to increasing the API’s permeation into malaria-infected erythrocytes, behavior that considerably diverges from that of the parent drug [149]. This trend was explained based on more pronounced electrostatic interactions of the charged anti-malarial drugs with the polar head groups of the phospholipids in the erythrocyte membranes, thus potentiating treatment efficacy [149].

The possibility of replacing the “inert” counterion of an API-IL by a second API may lead to dual therapeutic function ILs. However, the physicochemical and pharmaceutical properties of both APIs in the API-IL form, i.e., the resulting melting temperature, solubility, bioavailability, and stability will most likely be different for the two APIs in comparison to the precursors, and their adequate characterization is mandatory. Both lidocaine and etodolac, in the form of lidocainium etodolac ([Lid][Eto]), can exhibit superior water solubility than both APIs alone, with an increase of >90-fold for etodolac and two-fold for lidocaine [150]. Rogers and co-workers reported the enhancement of aqueous solubility, thermal stability and topical analgesic effect of lidocainium docusate ([Lid][Doc]) in comparison with the parent API (lidocaine hydrochloride)[129]. Lidocaine is known for its anesthetic effect, where docusate was selected due to its action as dispersing agent in formulations. Studies of anti-nociception in mice suggested that [Lid][Doc] produces a longer duration of antinociceptive effect than the original APIs. The longer pain relief afforded by this API-IL is provided by the synergistic effect of both APIs that impact pharmacokinetic, resulting in a different mechanism of action with higher therapeutic efficacy. The bioavailability of [Lid][Doc] was later addressed in vivo through its transdermal application on Sprague–Dawley rats [151]. The concentration of lidocaine in blood plasma was evaluated over time after the topical application of creams containing 5.0 wt% of [Lid][Doc]. However, the total systemic lidocaine absorption was almost undetectable even after 240 min. of the transdermal application. Despite the fact that many APIs display higher solubility in water and higher therapeutic efficacy when solubilized in ILs or when converted into ionic liquid salts, addressing the in vivo bioavailability of the developed formulations is a crucial requirement.

**Figure 13:**
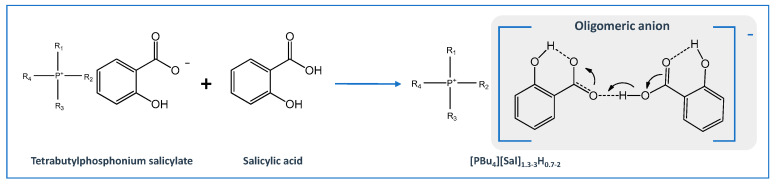
Schematic representation of the formation of oligomeric ions based on salicylate/salicylic acid proposed in [153].

The conversion of APIs into liquid forms as API-ILs may not always require a stochiometric approach. Oligomeric ion formation can be a possible alternative [152]. The preparation of oligomeric API-ILs, which are hydrogen-bonded moieties that include both the ions and the neutral non-ionized material, may contribute to the expansion of liquid drug formulations by simply changing the stoichiometry and/or complexity of the ions, i.e., by introducing the free acid/base of the conjugate base/acid within the salt formulation, thus lowering the API’s melting point [22]. This concept was first introduced in 2010, by Bica and coworkers [153], with tetrabutylphosphonium salicylates, ([PBu_4_][Sal]_nHm−1_). The combination of tetrabutylphosphonium hydroxide and salicylic acid beyond one equivalent originated several liquid compositions in a range of [PBu_4_][Sal]_1.3–3_H_0.7–2_, where the proton transfer is stronger. This behavior can be explained by the formation of hydrogen-bonded dimer complexes between salicylic acid and the salicylate anion, which can reach the composition of [PBu_4_][Sal]_3_H_2_ (Figure 13). MacFarlane et al. [154] presented a library of nine compounds and also four oligomeric API-ILs were synthesized and prepared. Benzoic, salicylic, and gentisic acids were chosen for this study, as these are frequently found in pharmaceutical formulations. These oligomers may modulate membrane transport properties in vivo, enabling a higher permeability of APIs in this form than the starting materials.

**Figure 14:**
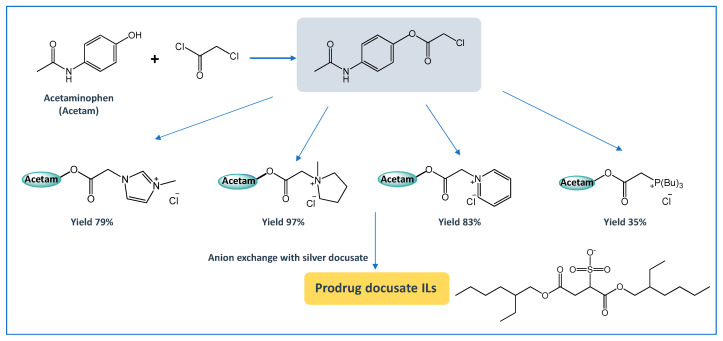
Synthesis of cationic acetaminophen prodrug ILs paired with the docusate anion proposed in [158].

Another way of enhancing the APIs therapeutic action and their delivery is by the prodrug approach. The prodrug concept has been refined over the years, and, nowadays, is defined by IUPAC as any compound that undergoes biotransformation before exhibiting pharmacological effect [155]. A prodrug only has activity after enzymatic and/or chemical transformation in vivo in order to release the active parent drug into the therapeutic target [156]. The prodrug strategy may enable the optimization of several drug properties, i.e., achieve higher stability, improved solubility and/or increased permeability. Yet, solid prodrugs can suffer from the same problems as any solid API and notably polymorphism [157]. Therefore, the combination of API-IL advantages with a prodrug strategy can be an appealing alternative to enhancing the APIs efficacy. The functionalization of APIs with easily biochemically cleavable (e.g., by hydrolysis) ionic functional moieties, which can be combined with selected counterions, is the basis of prodrug API-IL development. Similar to other IL strategies, it is possible to design the prodrug API-IL for a specific therapeutic purpose by the proper selection of the counterion, which may also include a second API (dual-function prodrug) or even a permeation enhancer [22,158]. A reported example of this approach encompasses liquid paracetamol-based drugs combined with imidazolium, pyrrolidinium, pyridinium, and phosphonium paired with docusate (Figure 14) [158]. The resulting prodrugs present lower water solubilities than the neutral paracetamol and slower release profiles in aqueous media. These differences in the paracetamol derivative properties may be advantageous for the development of controlled release systems. The promising results that were obtained with paracetamol may boost the study of this strategy for other APIs.

Despite being shortly explored, prodrug API-ILs can be designed to target specific tissues. Following this notion, a promising protecting-group-free synthesis for the modification of tertiary- and heteroaryl-amine containing complex small molecules with quaternary-ammonium linkers has recently been proposed as a chemoselective approach for targeted delivery [159].

The diversity in the API-IL toolbox stands as a unique possibility to provide new biologically active combinations that must be properly investigated. The largest fraction of published works focus on enhancing the APIs solubility; however, the assessment of in vitro and especially in vivo studies of API-ILs are still less reported. Furthermore, the lack of pharmacokinetic and pharmacodynamic studies with new API-ILs hinders the understanding of the changes on the therapeutic action, the metabolic pathways that are involved in their uptake and the alterations in toxicity in comparison with the precursor API. This gap might be partially attributed to the lack of guidelines from the pharmaceutical entities for API-ILs, which makes difficult their formulation and correct testing as new drugs. Establishing these lines will allow for overcoming these drawbacks and also considering other challenges, such as the scale-up, purification, stability, and delivery of liquid forms of therapeutics.

## 7. IL-Based Drug Delivery Systems

**Figure 15:**
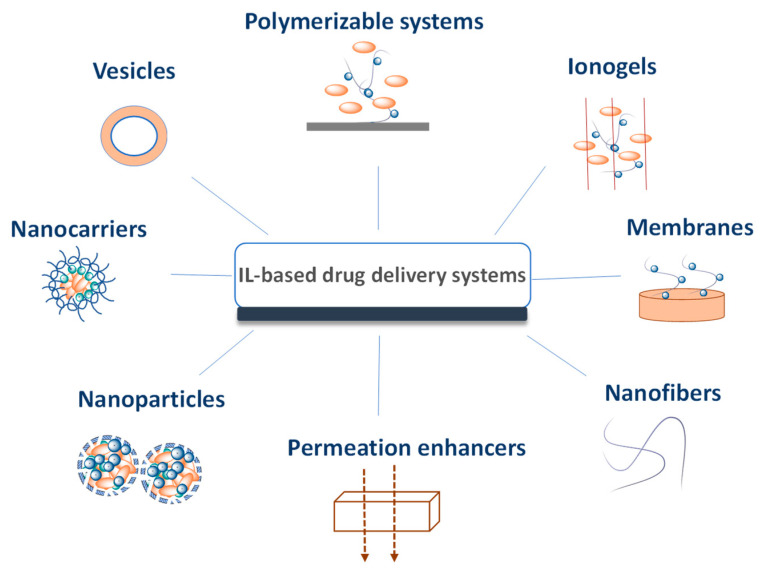
Schematic representation on the use of ILs in drug delivery systems (adequate references are given along the current section).

In the field of drug delivery, ILs have been applied as novel pharmaceutical forms (API-ILs) and the respective drug delivery appraised, and as solvents or as polymerizable monomers for the development of polymer drug delivery systems [75,79,80,160,161,162,163,164,165,166]. ILs are excellent solvents for a wide range of biopolymers, such as proteins [167,168], DNA [169,170], and polysaccharides [171,172,173], being used in their processing into films and micro and nanoparticles with potential for drug delivery. Additionally, ILs have shown promising ability to functionalize ionogels, opening new routes for designing advanced materials, including their use as drug release systems [174,175]. Figure 15 depicts the versatility of ILs in the development of novel drug delivery systems. Adding up to existing reviews on this area [30,161,176], our discussion focuses on the advances made for each type of administration route. The selection of the administration route is mostly dependent on the physicochemical properties of the API (e.g., melting temperature, molecular weight, polarity, solubility, etc.), its pharmacokinetic profile and ultimately the intended target site of action [6,177]. This selection is also dependent on other factors, such as invasiveness, where non-invasive routes of administration are preferable.

### 7.1. Intravenous Drug Delivery

Intravenous drug administration is a preferable choice when aiming to bypass biological absorption barriers [178]. The selection of this route offers several advantages, since it provides the most complete drug bioavailability with a minimal delay. Foreseeing intravenous drug delivery applications, ILs have recently been explored for the development of polymer nanocomplexes. In this field, the conjugation of SAILs with chitosan was studied [179]. The SAILs 1-butyl-3-methylimidazolium octylsulfate ([C_4_C_1_im][C_8_OSO_3_]) and 3-methyl-1-octylimidazolium chloride ([C_8_C_1_im]Cl) induced the formation of chitosan nanoparticles at low concentrations. While the negative charge of the counter ion Cl^-^ of [C_8_C_1_im]Cl could promote the IL-chitosan agglomeration (particles size of 450 nm), [C_4_C_1_im][C_8_OSO_3_] allowed for the formation of particles with smaller size due to stronger electrostatic interactions between the positively charged chitosan chain and [C_8_OSO_3_]^-^ ions (particles with 300 nm). Thus, work focused on the preparation and characterization of these particles; however, further studies regarding the encapsulation and release profile of a selected drug from these particles, as well as the knowledge of their cytotoxicity profile, are required issues to better appraise their potential in drug delivery. More recently, the IL 1-butyl-3-methylimidazolium acetate, ([C_4_C_1_im][CH_3_COO]), was applied as solvent media for the synthesis of an amphiphilic derivative of a chitosan oligosaccharide grafted with linoleic acid-(LCOS), followed by self-assembly in aqueous media [180]. The use of the IL allowed for a higher degree of grafting when compared to the values obtained with a conventional procedure using DMSO. It was then possible to obtain ibuprofen-loaded LCOS micelles in aqueous solution with an average size lower than 200 nm, suitable for intravenous administration. Despite the possibility to recover and reuse the IL from the reaction media, the cytotoxicity of the loaded LCOS micelles was not assessed.

**Figure 16:**
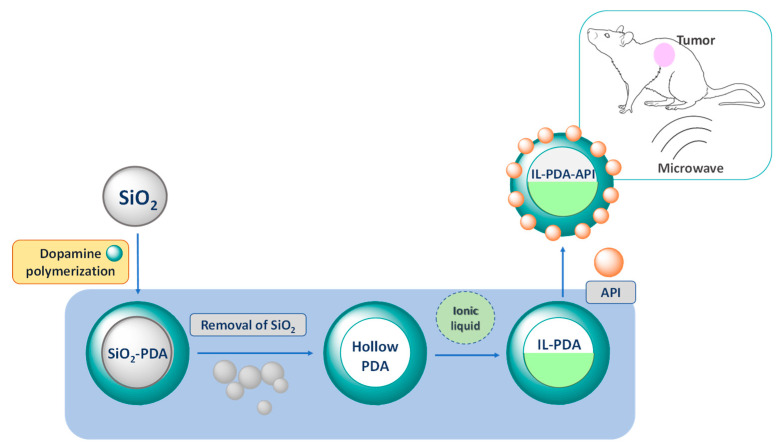
Schematic illustration of the preparation of IL-polydopamine (PDA) nanoparticles loaded with doxorrubucin for combined chemotherapy and microwave thermal therapy proposed in [181].

In a different approach, polydopamine nanoparticles that were loaded with doxorubicin and the IL [C_4_C_1_im][PF_6_] were recently developed for cancer treatment (Figure 16) [181]. The IL was employed as a microwave sensitizer to prepare these novel nanoplatforms for combined chemotherapy and microwave thermal therapy by intravenous administration. The antitumor efficacy of doxorubicin-loaded IL-polydopamine nanoparticles was demonstrated in in vitro and in vivo experiments in the treatment of tumors in mice, after intravenous injection via tail vein. The referred nanoparticles exhibited high inhibition effect when combined with the microwave thermal irradiation, acting in the tumor ablation without inducing significant tissue toxicity.

Micelles self-assembled from amphiphilic block copolymers were proposed for the administration of prednisone, a glucocorticoid that was mostly used to suppress the immune system [182]. To this end, the IL 1-allyl-3-methylimidazolium chloride ([(CH_2_CH=C_2_)C_1_im]Cl) was used as solvent in order to prepare cellulose grafted with polylactic acid by ring opening graft polymerization of l-lactide. Colloidal solutions comprising micelles of cellulose-g-PLLA were prepared in aqueous media by a membrane-dialysis method. The resulting micelles exhibited spheric morphology within a size range of 30–80 nm, and allowed for sustained drug release. The IL removal and the cytotoxicity evaluation in 3T3 mouse fibroblasts cell line showed low toxicity towards cells, reinforcing their potential applicability as drug carriers.

ILs can be used to form IL-in-oil (IL/O) [183], IL-in-water (IL/W) [79,184], oil-in-IL (O/IL), and water-in-IL (W/IL) [185] emulsions. The possibility to manipulate and design the IL structure allows for the use of IL-based vesicles and micelles as novel carriers of low-water soluble APIs. Accordingly, hydrophobic nontoxic ILs were used to prepare novel IL/W nanoemulsions for intravenous administration of amphotericin B [186]. Amphotericin B is an antifungal agent that, due to its low-water solubility (<1.0 μg mL^−1^) and self-aggregation in aqueous media, presents undesired side-effects, thus being its intravenous drug delivery a challenge. In a preliminary study, high contents (>5.0 mg mL^−1^) of the API were solubilized in a new hydrophobic dicholinium-based IL with the bis(trifluoromethanesulfonyl)imide ([NTf_2_]^−^) anion. The mixture of this hydrophobic IL with a hydrophilic cholinium-based IL resulted in the solubilization of the drug, preventing the concentration-dependent aggregation with controlled release of the API. Despite the maintenance of the antifungal activity of the API, and the low toxicity towards embryo-larval zebrafish models, further studies are required in order consider this formulation adequate for intravenous administration.

### 7.2. Oral Drug Delivery

The challenges that are faced in oral drug delivery are primarily related with the API’s poor bioavailability, i.e., related with the API’s dissolution, permeability, and solubility. An example of using ILs in the development of oral drug delivery systems comprises their application as monomers in the synthesis of positively charged polymers loaded with naproxen, in the form of an anionic API [187]. The drug delivery systems were prepared by free radical polymerization while using two IL monomers, 1-(4-vinylbenzyl)-3-methyl imidazolium hexafluorophosphate and 1-(4-vinylbenzyl)-4-(dimethylamino)-pyridinium hexafluorophosphate, and methyl styrene. The resulting positively charged polymers were loaded with naproxen and provided a controlled release of the API, avoiding the delivery in acidic and neutral media (pH 2–6.5). Given their pH-dependent behavior, these systems can be envisaged to target the intestine delivery. Similarly, ILs with a pH-sensitive character have been used in order to modify positively charged silica nanoparticles for oral delivery of methotrexate, a chemotherapeutic drug [188]. Imidazolium-based ILs were grafted to silica nanoparticles that were also grafted with polymethacrylic acid to form stimuli-responsive nanoparticles. These systems allowed for high drug encapsulation efficiencies (76%) due to the strong electrostatic interactions established between the nanoparticles and the API. Recently, cholinium geranate ([Ch][geranate_2_(H)]) has been used for insulin solubilization as a novel formulation for the administration of this API [189]. The IL-based insulin formulation was coated with Eudragit L-100 and orally administrated, exhibiting a promising, in vivo, pharmacokinetic and pharmacodynamic outcome. The respective oral bioavailability of the IL-insulin formulation was found to be 51% relative to subcutaneous injection of insulin. When orally administrated, this formulation significantly enhanced the paracellular transport of insulin, protecting it from enzymatic degradation, which resulted in a sustained decrease in blood glucose down to 45% in 2.5 h. This delivery system was also investigated for its cytotoxicity and stability, showing no relevant toxicity and a constant stability over two months at room temperature and for at least four months under refrigeration. Despite tolerability tests still being required to establish a direct comparison between this system and the injection administration of insulin, the possibility to develop alternative and less invasive routes of administration must be highlighted as an advantage of the use of ILs for delivery purposes. Additionally, this type of formulations preserves the API structure, avoiding immunological reactions or loss of the API in a multistep system development.

**Figure 17:**
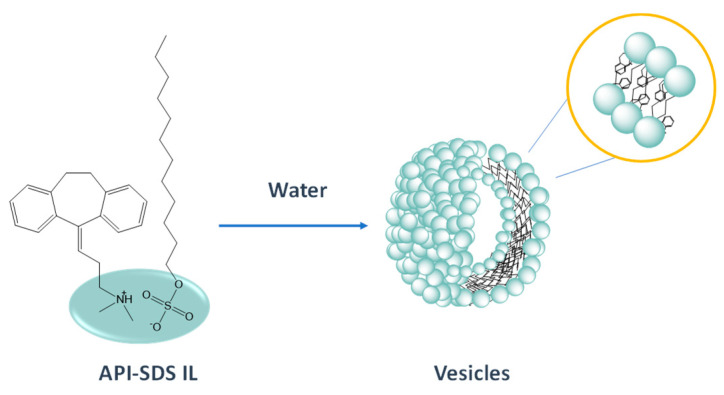
Illustration of the API-IL self-assembling into vesicles in aqueous media proposed in [191].

In a different approach, API-ILs have been incorporated in carrier materials for the development of IL-based oral drug delivery systems. For instance, tetrabutylphosphonium ibuprofenate ([P_4444_][Ibu]) and lidocainium ibuprofenate ([Lid][Ibu]) were successfully immobilized into mesoporous silica particles for a fast and complete API release when placed into an aqueous environment, where approximately within 5 min. all IL was released from the solid support [190]. Zhang et al. [191] proposed an all-in-one concept for the application of API-ILs as drug delivery systems. The pharmaceutically active IL itself works as both the carrier and the active drug. The API-IL self-assemble into vesicles in aqueous solution due to the combination of an anionic surfactant (sodium dodecylsulfate) with a cationic drug with anti-depressive properties (amitriptyline hydrochloride) (Figure 17). Furthermore, the referred IL-based vesicles have high drug loading contents, an advantage over conventional drug delivery systems. Also, it was possible to control the release profile of the API, moving from a total release of 74% and 82% to 28% and 32% in just 2 h, using the correspondent API-IL, at pH 7.4 and pH 1.2, respectively.

Itraconazole and cinnarizine were converted into lipophilic ILs (cinnarizine decylsulfate, itraconazole dioctyl sulfosuccinate) aiming to facilitate their incorporation into lipid-based formulations [192]. The resulting API−ILs were completely miscible or highly soluble in lipid-based self-emulsifying drug delivery systems (SEDDs). These systems, composed of long or medium chain glycerides, surfactants, such as Kolliphor-EL, and cosolvents, like ethanol, were easily incorporated into lipid-based formulations for in vivo oral drug delivery. The pharmacokinetic evaluation upon the administration of SEDDs revealed higher drug plasma exposure for the API-IL formulations (2-fold for cinnarizine and 20-fold for itraconazole) in comparison with the SEDDs with the respective parent APIs. The use of API-ILs for the development of these formulations enabled obtaining liquid SEDDs, increasing the oral exposure to the API. The increase in the drug absorption is enabled by the increase in the APIs’ solubility and by promoting the gastrointestinal lipid absorption pathways. The design of the IL structure was also employed for optimization of the solubility of danazol, an API used in the treatment of endometriosis and fibrocystic breast disease, and itraconazole, an antifungal drug [77]. Like many other low-water soluble APIs, itraconazole and danazol present low-water and lipidic solubilities. Such conditions hinder the selection of formulation excipients that can enhance these drugs’ bioavailability. The solubility of danazol and itraconazole was increased 20-fold and >500-fold using 1-hexyl-3-hexyloxycarbonylpyridinium dicyanamide ([C_6_C_6_OCOpy][N(CN)_2_]), when compared to its solubility in soybean oil (a common lipid excipient). These solubility enhancements surpass those that were provided by using co-solvents like PEG and ethanol. The oral administration of the danazol-containing self-emulsifying IL formulation rises up to 4.3-fold the API’s bioavailability when compared to the respective crystalline drug. Not only the absorption of the API was improved, but also its release sustained, as the formulation prolonged the plasma exposure to the API when compared with the respective lipid formulation.

### 7.3. Topical and Transdermal Drug Delivery

**Figure 18:**
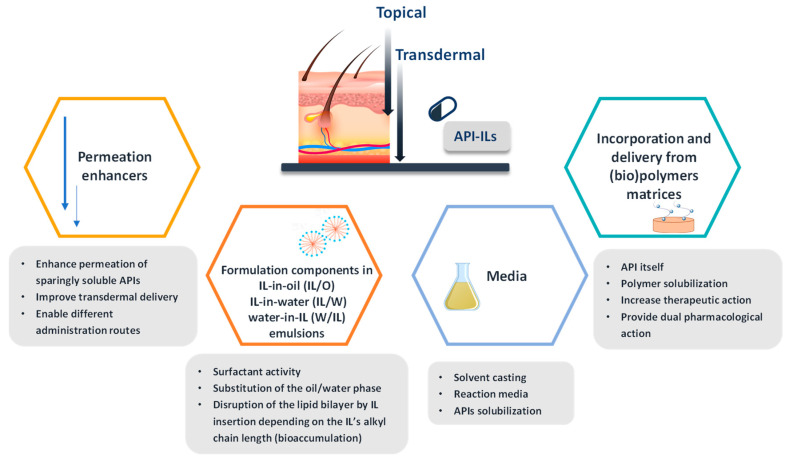
IL applications in the development of drug delivery systems for topical and transdermal administration (adequate references are given along the current section).

When considering the topical application of APIs, the drug delivery system must target one or more different skin layers and underlying tissues, or skin associated structures (sebaceous or sweat glands, etc.) [193]. Transdermal drug delivery, in particular, aims to reach systemic circulation, representing an alternative to parenteral and oral routes, while avoiding pre-systemic metabolism [194]. Despite being just recently explored, ILs have been studied as promising pharmaceutical agents or formulation components in order to tackle the challenges in topical and transdermal delivery systems, presenting a wide range of applications, as illustrated in Figure 18 [195]. ILs have been investigated in microemulsions, nanoparticles, (bio)polymer-based drug delivery systems (e.g., patches and membranes), and as permeation enhancers aiming to deliver poorly water-soluble drugs at topical and transdermal level, as summarized in Table 3.

Microemulsions have been proposed in order to improve transdermal delivery, especially when comprising ILs. A single IL can replace different components in a given drug formulation, by substituting the oil, water or surfactant phase in microemulsions, acting as the permeation enhancer and/or being the API itself, as aforementioned. Furthermore, the fine-tuning of SAILs allows for manipulating the structure and dynamics of their micellar aggregates, making these ILs promising vehicles for APIs delivery due to their ability to enhance the solubility and specially the permeability of the drug across biological membranes.

The design of IL-excipient with tunable lipophilicity/hydrophilicity character is advantageous, especially when used as a solubility-enhancing agent for complex amphiphilic APIs, like amphotericin B and itraconazole, which are antifungal drugs [76]. Mahajan et al. [196] studied the performance of the SAIL 1-methyl-3-tetradecylimidazolium bromide ([C_14_C_1_im]Br) as a drug carrier, and then compared it to a conventional cationic surfactant tetradecyltrimethylammonium bromide (TTAB). [C_14_C_1_im]Br revealed not only superior surface activity, but also acts as better drug carrier of APIs, namely dopamine hydrochloride and acetylcholine chloride, than the traditional TTAB. Further, Sanan et al. [87] established the effect of the aggregate morphology and dilution on the micellar transition of [C_12_C_1_im]Cl and ibuprofen mixtures. The transition is driven by the release of the API from the mixed micelles due to the solubility disparity between both components. Similar SAILs, [C_12_C_1_im]Cl and 1-methyl-3-tetradecylimidazolium chloride ([C_14_C_1_im]Cl) have shown capability to form aggregates with lidocaine hydrochloride, improving the drug’s dissolution into aqueous media [197]. Furthermore, the reported thermal stability of nonaqueous IL microemulsions (up to 150 °C) was shown to be compatible with sterilization processes, an advantage over conventional formulations [212]. All of the described works envisioned the application of the studied emulsions in transdermal and topical drug delivery; however, permeation studies on skin models were not conducted for evaluating the ability of these systems to improve dermal delivery.

The influence of imidazolium-based ILs on the properties and stability of oil-in-water (W/O) and water-in-oil (O/W) emulsions was investigated by Dobler et al. [29] using a fluorescent probe, namely Reichardt’s dye, as a drug model. A hydrophilic IL, 1-hexyl-3-methylimidazolium chloride ([C_6_C_1_im]Cl), and a hydrophobic IL, [C_4_C_1_im][PF_6_], were incorporated as water and oil phase components, respectively, resulting in stable formulations. Skin permeation across pig’s ear skin was studied in vitro on Franz glass diffusion cells. A permeation enhancement was observed when using these formulations due to the disruption of the lipidic bilayer packing by the ILs used. These ILs present antimicrobial activity and preservative efficacy within these formulations, being possible to fulfil the requirements as novel preservatives. Similar ILs where studied for an IL/W microemulsion designed for etodolac’s topical delivery [79], which is used in the treatment of inflammation and pain that are associated with rheumatoid arthritis and osteoarthritis [213]. The prepared microemulsion was based on [C_4_C_1_im][PF_6_], Tween 80 as surfactant, and ethanol as co-surfactant. The IL/W-based formulation was able to efficiently enhance the solubility and ex-vivo permeability of the API for its transdermal delivery [79]. Skin penetration was evaluated while using the fluorescent dyes sodium fluorescein (a hydrophilic fluorescence marker) and Nile red (lipophilic), which in the presence of ILs revealed a more efficient penetration into the deeper skin layers. Likewise, in-vivo pharmacodynamic evaluation showed improved anti-arthritic and anti-inflammatory activities in comparison to O/W microemulsions and marketed etodolac’s formulations, exhibiting the high capability of these formulationsin order to enhance the API’s performance.

**Figure 19:**
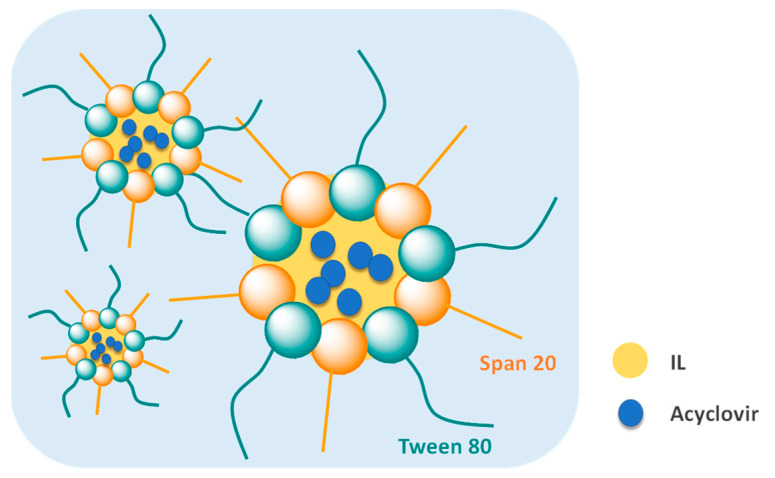
IL-in-oil microemulsions based on [C_1_C_1_im][(MeO)_2_PO_2_] as drug carriers of acyclovir proposed in [198].

The first IL-based microemulsion reported for transdermal delivery aimed to improve membrane transport of a sparingly soluble API, namely the antiviral drug acyclovir [198]. In this work, a blend of two nontoxic surfactants, Tween-80 and Span-20, was used in combination with imidazolium-based ILs to form stabilized IL droplets. In the referred microemulsion, the external phase (oil phase) is constituted by isopropyl myristate (Figure 19). Among the investigated ILs, dimethylimidazolium dimethylphosphate ([C_1_C_1_im][(CH_3_O)_2_PO_2_]) presented superior ability to dissolve the selected API and form more stable droplets in the formulation. This improvement was justified by the hydrogen bonding interactions between the polar groups of acyclovir and the IL anions. The in vitro study across Yucatan micropig porcine skin (performed on Franz diffusion cells), allowed for verifying an increase in acyclovir’s skin permeability of several orders of magnitude, as well as the API’s transdermal permeation when using the IL/O system as drug carrier.

Cholinium-based ILs comprising anions that were derived from carboxylic acids were also used in IL/O microemulsions to increase the transdermal delivery of acyclovir, as non-toxic and non-irritating alternatives [199]. Hydrophilic ILs (cholinium formate, cholinium lactate and cholinium propionate) were used as the non-aqueous polar phase and a surface-active IL (cholinium oleate) as the surfactant in combination with a co-surfactant, Span 20, in a continuous oil phase. An enhancement in skin permeation due to the modification and disruption of the regular arrangement of the corneocytes of the *stratum corneum* was observed, and related to the ionic character of the IL. Cytotoxicity tests revealed a high cell survival rate (>90%) in comparison with Dulbecco’s phosphate-buffered saline solution, highlighting the potential of these formulations as low toxic drug carriers. Similarly, the IL [C_1_C_1_im][(CH_3_O)_2_PO_2_] was used in an IL/O microemulsion in order to improve the transdermal delivery of the sparingly soluble chemotherapeutic methotrexate [200]. In this work, drug permeation across a similar skin model to the previous enounced was evaluated, revealing a significant transdermal permeation of the API in comparison to the application of other typical O/W and O/W formulations. IL-based microemulsions were also developed for enhancing the skin permeation of dencichine, a haemostatic agent [201]. An initial screening with fourteen imidazolium-based ILs was performed, from which 1-(2-hydroxyethyl)-3-methylimidazolium chloride ([C_2_OHC_1_im]Cl) and dimethylimidazolium dodecanesulfate ([C_1_C_1_im][C_12_SO_3_]) were selected and incorporated into the aqueous and surfactant phases, respectively, with an enhancement on skin permeation of approximately 10-fold. However, despite that the in vivo pharmacodynamic activity was found to be in good correlation with the in vitro permeability, hemostatic activity studies revealed no statistic difference between these formulations and dencichine aqueous solution.

**Figure 20:**
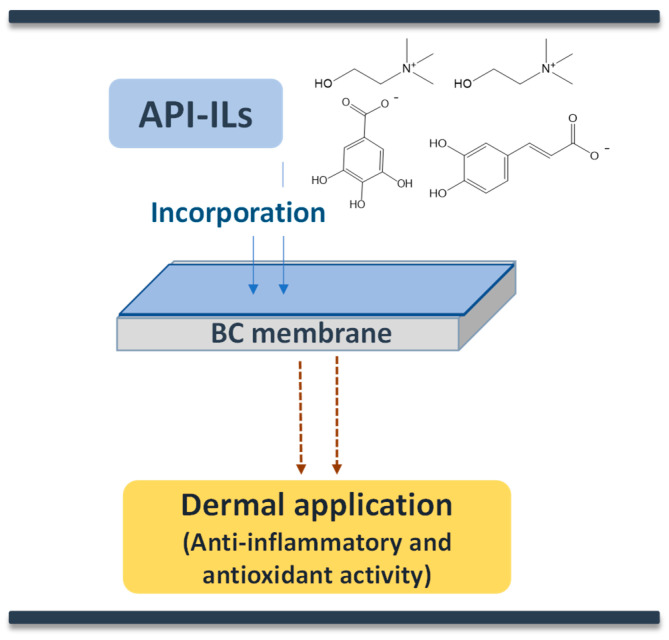
Illustration of BC-IL membranes’ with antioxidant and anti-inflammatory action for dermal application proposed in [202].

The application of API-ILs represents an advantage in the design of topical delivery systems due not only to the possibility of providing new biologically active combinations and enhancing the therapeutic action, but also due to the possibility to enhance the transdermal delivery of pharmaceuticals. The use of API-ILs might allow significant developments in delivery systems, since these can be designed to self-aggregate, trapping the drug in a micelle, or to display tunable hydrophilic-lipophilic balance to potentiate the drugs permeation. In addition to the incorporation of ILs into microemulsions as transdermal drug delivery systems, the combination of API-ILs with polymers and biopolymers has been recently investigated. Morais et al. [202] synthesized cholinium-based ILs paired with anions that are derived from phenolic acids, namely gallic, caffeic, and ellagic acids. These ILs were incorporated into bacterial nanocellulose membranes (BC). The developed drug delivery systems showed superior antioxidant activity to the starting materials, and with controlled diffusion of the active compounds from the wet membranes. The obtained results demonstrated that the dissolution profiles were essentially governed by the solubility of the ILs rather by their interactions with the BC nanofibrils. For both BC-[Ch][Caf] and BC-[Ch][Gal] wet membranes, approximately 70% dissolution of the IL content in membranes was reached after 6 h. Regarding the cytotoxicity of these delivery systems, it was shown that they do not cause any decrease in cell viability at the concentrations investigated. Additionally, the exposure of cells to BC-ILs membranes significantly decreases the LPS-induced NO production, indicating a relevant anti-inflammatory and antioxidant potential (Figure 20). The permeation flux of both API-ILs from the BC membranes was assessed in vitro in human epidermal skin, at body’s temperature, on Franz diffusion cells. The skin permeation assay showed the possibility to obtain a slow and sustained release profile while using these drug delivery systems over 5 h of administration.

Chantereau et al. [203] later incorporated NSAID-based ILs also into BC membranes envisaging their use in transdermal drug delivery systems. These [Ch][NSAID] ILs allowed for increasing up to 100-fold the solubility of the respective NSAID precursors (ibuprofen, naproxen and ketoprofen) in aqueous media. The impregnation of BC membranes with these ILs also increased, by 18 to 26-fold, the rehydration ability of the membranes, allowing their potential application on the absorption of exudates. Giving the obtained results, the developed systems are promising alternatives for the design of transdermal patches for anti-inflammatory drugs delivery. More recently, the same researchers investigated the possibility to develop membranes that were loaded with vitamin B-based ILs for dermal applications [204]. Three ILs, namely cholinium nicotinate ([Ch][B3]), cholinium pantothenate ([Ch][B5]), and cholinium pyridoxylate ([Ch][B6]), were also synthesized and incorporated in BC, resulting in more thermally stable forms for the vitamins without toxicity associated for skin cells. The solubility of these ILs in aqueous media was higher than their vitamin precursors, with solubility enhancements up to 30.6-fold. The increase on the re-hydration ability of BC-IL membranes, allowed for obtaining a complete and faster release profile of ILs in aqueous media than the release of the precursor vitamins. These ILs also displayed a plasticizing effect on the BC membranes, favoring the application of these systems on irregular skin regions.

The IL [Lid][Eto] has been incorporated into a topical delivery system [205]. The patch (Etoreat), studied by IL Pharma Inc. (MEDRx, Kagawa, Japan), contains the only API-IL that has reached clinical trials. This API-IL based patch for alleviating pain caused by inflammation was tested for the treatment of ankle sprains and low back pain, due to its enhanced skin absorption. However, due to the lack of statistic significant results between Etoreat and placebo administration, its further stage of development was suspended. In addition to this example, other dual-active API-ILs have been considered for improving transdermal delivery of analgesic and anti-inflammatory APIs in wound healing [206]. Lidocainium naproxen ([Lid][Nap]), [Lid][Ibu], and lidocainium diclofenac ([Lid][Dicl]) were synthesized, which resulted in liquid mixtures at room temperature. These API-ILs displayed significantly higher solubility in aqueous media than the parent APIs, except for [Lid][Dicl]. These ILs were then incorporated into a bilayer wound dressing, which was composed of a hydrophobic polyvinylidene fluoride membrane that acts as a drug reservoir and a biocompatible hyaluronic acid layer. The assessment of anti-inflammatory activity revealed similar therapeutic efficacy when compared with the original APIs, through the inhibition of LPS-induced production of nitric oxide and prostaglandin E2 by macrophages. These systems enable higher permeation of both APIs in API-IL form than the parent APIs without compromising the fibroblasts proliferation. Furthermore, the hyaluronic acid that was used in these systems played a protective effect on the cytotoxicity since it minimized the potential antiproliferative effects attributable to the APIs, allowing the simultaneous delivery of anti-inflammatory and analgesic drugs to the injured area without compromising skin regeneration.

ILs were also studied as solubility enhancers in conjugation with polymers as novel nanoparticle hybrid systems for the delivery of poorly water-soluble drugs [207]. Two amino-acid-based ILs, namely cholinium phenylalanine ([Ch][Phe]) and cholinium L-glutamine ([Ch][Glu]), were used in blends with poly-(lactic-co-glycolic acid) (PLGA) that was loaded with rutin, which shows antidiabetic, antihypertensive, and antilipidemic activities. These systems allowed for a drug loading capacity higher than 50% for both ILs, and up to 76% for [Ch][Phe] while using only 0.2% (v/v) of IL. The systems provided a sustained release of rutin, with 85% released after 72 h, without toxicity to associated skin cells.

**Figure 21:**
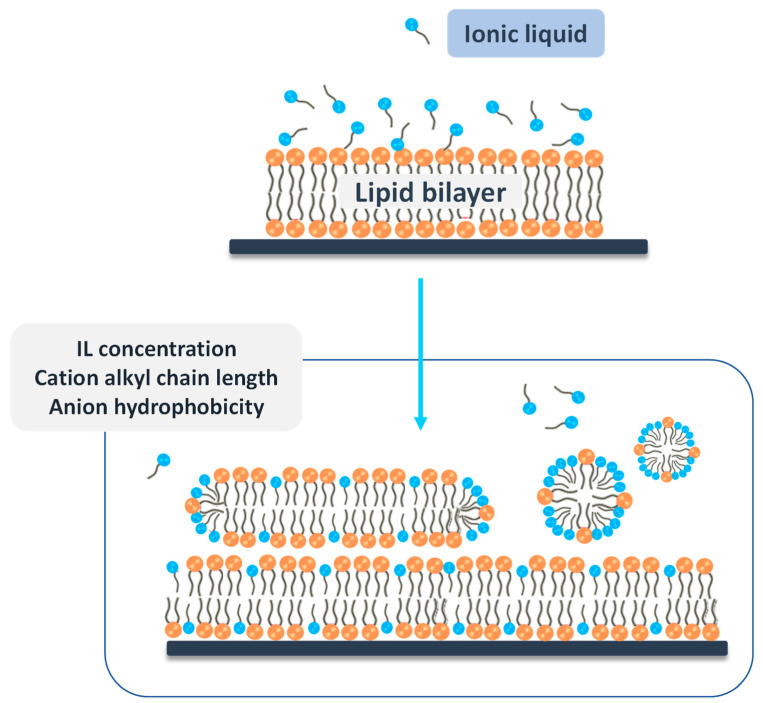
Schematic representation of IL effect and insertion into the lipid bilayer proposed in [214].

Zhang et al. [208] used testosterone as a model drug to investigate the transdermal delivery enhancement provided by twenty imidazolium-based ILs. The conducted study revealed an interdependence between the API permeation enhancement and the structure and composition of the IL. ILs with longer alkyl side chains (N-octylimidazolium chloride ([C_8_im]Cl)), 1-octyl-3-methyl imidazolium chloride ([C_1_C_8_im]Cl) and 1-octyl-2,3-dimethyl imidazolium chloride ([C_1_C_1_C_8_im]Cl)) led to higher transdermal delivery enhancements. Additionally, the number of alkyl groups at the cation, as well as the anionic constitution, was demonstrated to have an impact on the drug penetration through skin. However, this trend follows the cytotoxicity profile and disruptive character of ILs with longer alkyl chain lengths, in which the most cytotoxic compromise in a larger extent the structural integrity of biological membranes. The enhancement of API permeation was attributed to the change in skin permeability, rather than the change in drug concentration. Evaluations by ATR-FTIR and atomic force microscopy of skin membrane indicated that the ILs can disrupt the regular and compact arrangements of the corneocytes, altering the skin structure to a more permeable state. Although this mechanism was not observed for all ILs, further information must be gathered in order to properly understand the process of the interaction between these ILs and skin cells to better design IL-based permeation enhancers. This subject has been addressed by Jing and coworkers [214], which confirmed that amphiphilic ILs could disrupt the lipid bilayer by IL insertion, endcapping the hydrophobic edge of the lipid bilayer, and eventually disintegrating the membrane (Figure 21). This destabilization is directly related with the IL concentration, the length of the IL cation alkyl chain, and anion hydrophobicity, which are also correlated with the IL cytotoxicity. Hydrophilic and hydrophobic ILs seem to act on the APIs transportation through the stratum corneum by different mechanisms [215]. While hydrophilic ILs, particularly imidazolium-based ones, fluidize the cell membrane in order to create pathways for the diffusion of molecules (paracellular transport), hydrophobic ILs, on the contrary, modify the APIs partitioning by providing channels through biological membranes (transcellular transport) [211,216].

Different ILs that were based on carboxylic acids [217], aliphatic amines, amino acids [209,218] and polyethylene glycol derivatives [210] have been also studied as alternative permeation enhancers. An amino acid ester (proline ethylester) was used in combination with ibuprofen as a novel API-IL (proline ethylester ibuprofenate ([ProOEt][Ibu])) to improve transdermal delivery [209]. An approximately 10-fold enhancement in the cumulative amount of API was achieved at 96 h when compared with the control sample while using [ProOEt][Ibu].

ILs generated by a neutralization reaction between aliphatic carboxylic acids (octanoic acid or isostearic acid) and aliphatic amines (diisopropanolamine or triisopropanolamine) were proposed in order to study the mechanism of permeability enhancement of model hydrophilic and hydrophobic APIs [217]. The model formulation containing these ILs exhibited a more pronounced permeation enhancement under acidic excess conditions than under neutral environments. Despite that these formulations displayed superior controlled release for the hydrophilic model API, the mechanism that was responsible for this behavior was not further explored. Later, the difference between API-ILs transport across membranes and the respective commercial sodium salts was studied for salicylic acid [210]. The membrane transport ability and rate of the ILs triethylene glycol monomethyl ether tributylammonium salicylate ([mPEG_3_N_444_][Sal]), tributylammonium salicylate ([HN_444_][Sal]), cholinium salicylate ([Ch][Sal]), and 1-methylpyrrolidiniumsalicylate ([C_1_Pyrr][Sal]), as well as the respective sodium salt (Na[Sal]) and neutral form (HSal), was evaluated while using a Franz diffusion cell system. The cation influence in the efficiency of the membrane transport was also highlighted, as [mPEG_3_N_444_][Sal] showed a transport enhancement by ~2.5-fold in comparison to PEG-free cations.

Zakrewsky et al. [211] investigated the application of ILs in a multiple context for drug delivery, where the IL [Ch][geranate_2_(H)] was used not only to improve transdermal delivery, but also to tackle skin biofilm-protected microbial infections. This IL allowed to increase by 16-fold the delivery of a model antibiotic, cefadroxil, into deeper skin layers when compared to its aqueous solution. The potential clinical efficacy of the IL formulation was accessed in vivo based on its antimicrobial activity against biofilm-infected wounds. [Ch][geranate_2_(H)] enabled reducing *Pseudomonas aeruginosa* and *Salmonella enterica* bacterial viability by >95% after 2 h. The IL ability to disrupt the bacterial biofilm allowed for delivering the antibiotic with increased efficacy, improving the pathogens susceptibility to the antibiotic.

For topical and transdermal, as it happens for intravenous and oral delivery systems, it is important to understand and better evaluate the activity, in vivo behavior, and safety to achieve more effective and less toxic options with a desired drug activity. Although an increase in in vivo results on the administration of IL-based formulations has been observed, the available information is still scarce. The evaluation of pharmacokinetic and pharmacodynamic parameters as well as the therapeutic efficacy must be encouraged to be pursued to provide missing insights on this strategy. In the future, other IL-(bio)polymer combinations are expected and the understanding of their mechanistic levels should be encouraged to be unveiled. The understanding of these therapeutic options and the increase in the research of ILs in the nanoparticle field will allow for developing targeted-specific drug delivery systems that will reduce drug side-effects and fluctuation in circulating drug levels, optimizing the treatment efficacy. For this, ILs use in drug delivery should be further explored from the use of imidazolium-based ILs to options with low toxicity and known cytotoxicity profiles. Additionally, it is expected an increase in the studies comprising IL-(bio)polymer-based systems with stimulus-responsiveness, and investigation on their ability to protect drugs from degradation while providing controlled drug release.

## 8. Conclusions and Future Perspectives

Organic volatile solvents have been a main choice in the pharmaceutical industry, particularly in the reaction and purification steps, but they still raise concerns on the contamination of the final product and on the related environmental and health impacts. To overcome some of these drawbacks, ILs have been investigated as solvents, reagents, or catalysts in the synthesis of APIs and applied in the crystallization process of drugs. Still, additional studies should focus on the development of more sustainable strategies to remove ILs after the APIs synthesis. A careful monitorization of these contaminants in the final product and study of their impact in the drug’s performance and toxicity must be taking into consideration. Furthermore, the use of solvents and co-solvents, like ethanol, methanol or DMSO, hydrotropes, and surface-active agents, to improve the API’s solubility in pharmaceutical formulations has been challenged by applying ILs to this purpose. ILs have been shown to allow the aqueous solubility improvement of APIs from distinct pharmacological classes in several orders of magnitude (to be best of our knowledge and up to date, up to 5.6 × 10^6^-fold), standing as competitive alternatives to organic solvents. However, these formulations require a more comprehensive study in what concerns the stability, absorption and bioavailability of APIs. Furthermore, more recent studies employing aqueous solutions of ILs instead of pure ILs can be the key for their use and acceptance by the pharmaceutical field.

Because the APIs’ solubility in aqueous solution and bioavailability can be limited by polymorphism, controlling this process is essential for obtaining a stable and high-quality drug product. The study of ILs in the crystallization process of APIs has enabled the possibility to design new polymorphs, with higher thermal stability, to select the crystal form and habit, and even isolate and purify the correct API polymorph through crystallization. To expand the research on this topic, it is still mandatory to comprehensively understand the IL-API interactions that drive the formation of specific polymorphic forms and habits. The use of computational tools can be helpful in designing ILs in such a way. Furthermore, as it happens with the APIs synthesis, the research on effective separation methods and the limitation of the IL contamination in the final product are highly demanding issues.

ILs can be designed to present biological activities due to the broad number of anion-cation combinations. In this sense, ILs have been successfully studied regarding their antimicrobial, antioxidant, and anti-tumoral activities. So far, IL activities have been mainly studied in vitro and have focused on imidazolium-based ILs. To expand this field of research, it is necessary to unveil the mechanisms of interaction between the IL and biological membranes and, consequently, establish a correlation with their biological activities, and in which computational tools may also play a crucial role. The possibility to manipulate the cation-anion combinations also allowed for obtaining new drugs with desired chemical and biological properties, while avoiding polymorphism concerns. API-ILs have provided double action in therapeutic formulations for topical and transdermal delivery, namely by providing the API facilitating its permeation through biological membranes. In this field, API-ILs stand as novel liquid forms that can be designed with a specific or dual pharmacological action, obtained by incorporating APIs as IL ions, using oligomeric ions or by applying a prodrug strategy. However, in this field, only few works conducted bioavailability assays, however allowing to demonstrate the increase in the API’s bioavailability and the therapeutic efficacy of these novel drugs. Because API-ILs can present contrastive, enhanced or even dual effect when compared to the initial precursors, in vivo pharmacokinetic and pharmacodynamic tests are mandatory in understanding the pathways that are involved in their absorption, metabolism, and routes of elimination. These assays are required to enable the acceptance of these new drugs by the pharmaceutical industry, favoring the establishment of guidelines for their development and research. Until their implementation, additional obstacles are expected to be faced. The pharmaceutical industry is majorly prepared in order to produce solid APIs; thus, the scale-up implementation might be a lengthy process. Years of routinely working with solid forms of APIs might make the development of standardized procedures for the liquid drugs’ purification difficult.

The flexibility of ILs allowed for the development of tailored (bio)polymer drug delivery systems as well, both due to their polymerizable character and polymer solvation ability. Because ILs can enhance the APIs solubility in aqueous media, they have successfully allowed the incorporation and delivery of several low-water soluble drugs, enabling the consideration of new administration routes. However, the lack of more complete studies on this topic that can assist the conscious development of more effective drug delivery options still confines their use and commercialization. Advances in this area should comprise integrated studies where the IL can be designed with a specific biological activity and/or therapeutic action. These designed ILs can simultaneously have a specific role in the development of the drug delivery systems. In this line, stimuli-responsive drug delivery systems, promoted by the IL and/or by the polymer, also are of particular relevance.

Overall, ILs have potential to overcome solubility, bioavailability, permeation, polymorphism, and stability concerns that are associated to solid-state pharmaceuticals. Furthermore, ILs are promising alternatives to volatile organic solvents when applied as solvents, reagents, and anti-solvents in the synthesis and crystallization of active pharmaceutical ingredients (APIs). More recent studies demonstrated their potential to improve the performance of drug-delivery-based systems. The results and advances herein revised support the multiple roles of ILs in the pharmaceutical field, encouraging new ways of taking advantage of their unique properties.

## Figures and Tables

**Table 1:** Solubility of different APIs in selected ILs and comparison with their water solubility.

API	Structure	Water Solubility	IL	Solubility	Reference
4-Hydroxycoumarin	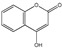	-	[P_666(14)_][NTf_2_]	0.0524 ^a^ *	[72]
			[C_2_C_1_im][CF_3_O_3_S]	0.1107 ^a^ *	
			[C_4_C_1_im][CF_3_O_3_S]	0.0907 ^a^ *	[73]
5-Fluorouracil	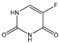	12.21 ^b^ *	[C_4_C_1_im]Br	31.19 ^b^ *	[74]
Acetaminophen	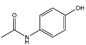	98.8 ^c^19.16 ^b^	[C_4_C_1_im][BF_4_]	>132 ^c^	[69]
			[C_8_C_1_im][BF_4_]	126 ^c^	
			[C_4_C_1_im][PF_6_]	52 ^c^	
			[C_8_C_1_im][PF_6_]	10 ^c^	
			[C_6_C_1_im][PF_6_]	13.21 ^b^	[75]
Acetylcysteine	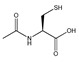	-	[C_2_C_1_im][CF_3_O_3_S]	0.1711^a^ *	[73]
			[C_4_C_1_im][CF_3_O_3_S]	0.1088 ^a^ *	
			[C_4_C_1_im][NTf_2_]	0.0866 ^a^ *	
			[C_6_C_1_im][NTf_2_]	0.0635 ^a^	
			[C_10_C_1_im][NTf_2_]	0.0102 ^a^ *	
Albendazole	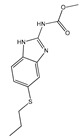	0.0020 ^c^	[C_4_C_1_im][BF_4_]	1.49 ^c^	[69]
			[C_6_C_1_im][BF_4_]	2.97 ^c^	
			[C_8_C_1_im] [BF_4_]	7.2 ^c^	
			[C_4_C_1_im] [PF_6_]	29 ^c^	
			[C_6_C_1_im] [PF_6_]	53 ^c^	
			[C_8_C_1_im] [PF_6_]	>75 ^c^	
Amphotericin B	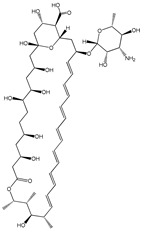	2.0 × 10^−4 b^	[C_2_C_1_im][CH_3_COO]	85 ^b^	[76]
			[C_4_NH_3_][CH_3_COO]	30 ^b^	
			[C_6_NH_3_][CH_3_COO]	30 ^b^	
			[C_8_NH_3_][CH_3_COO]	20 ^b^	
			[C_4_NH_3_][Oleate]	<5 ^b^	
			[C_6_NH_3_][Oleate]	<5 ^b^	
			[C_8_NH_3_][Oleate]	<5 ^b^	
Danazol	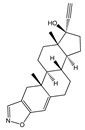	0.00030 ^c^	[C_4_C_1_im] [BF_4_]	18.9 ^c^	[69]
			[C_8_C_1_im] [BF_4_]	>59 ^c^	
			[C_4_C_1_im] [PF_6_]	11.9 ^c^	
			[C_8_C_1_im] [PF_6_]	35 ^c^	
			[C_6_C_6_OCOpy][N(CN)_2_	>90 ^d^	[77]
			[C_6_C_6_OCOpy][NTf_2_]	25 ^d^	
Erythromycin	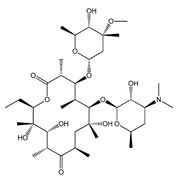	-	[C_4_C_1_im] [NTf_2_]	0.037 ^a^ *	[78]
			[C_10_C_1_im] [NTf_2_]	0.072 ^a^	
			[P_666(14)_]Cl	0.085 ^a^	
			[N_4,1,1,1_][NTf_2_]	0.053 ^a^	
			[Pyrr_4,1_][NTf_2_]	0.017 ^a^	
Etodolac	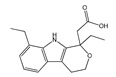	Insoluble	[C_4_C_1_im] [PF_6_]	374.33 ^b^ *	[79]
Fenofibrate	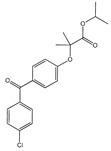	-	[C_6_C_6_OCOpy][N(CN)_2_]	>125 ^d^	[77]
			[C_6_C_6_OCOpy][NTf_2_]	>130 ^d^	
Glibenclamide	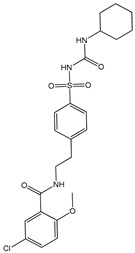	2.4 × 10^−6 b^ *	[Ch][Try]	9.89 ^b^ *	[80]
Ibuprofen	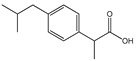	0.124	[C_4_C_1_im] [PF_6_]	6.95 ^b^	[75]
			[C_6_C_1_im] [PF_6_]	26.38 ^b^	
			[P_666(14)_][NTf_2_]	0.0528 ^a^	[72]
Isoniazid	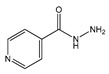	-	[DDA][NO_3_]	0.0452 ^a^ *	[81]
			[C_2_][NTf_2_]	0.0235 ^a^	[82]
			[C_4_C_1_im][NTf2]	0.004 ^c^	
			[C_6_C_1_im][NTf2]	0.003 ^c^	
			[P_666(14)_][NTf_2_]	0.0651 ^c^	[72]
Itraconazole	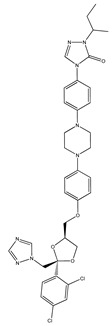	1.0 × 10^−6 b^	[C_2_C_1_im][CH_3_COO]	<5 ^b^	[76]
			[C_4_NH_3_][CH_3_COO]	<5 ^b^	
			[C_6_NH_3_][CH_3_COO]	<5 ^b^	
			[C_8_NH_3_][CH_3_COO]	<5 ^b^	
			[C_4_NH_3_][Oleate]	<5 ^b^	
			[C_6_NH_3_][Oleate]	<5 ^b^	
			[C_8_NH_3_][Oleate]	<5 ^b^	
			[C_6_C_6_OCOpy][N(CN)_2_]	40 ^d^	[77]
		-	[C_6_C_6_OCOpy][NTf_2_]	8 ^d^	
Paclitaxel	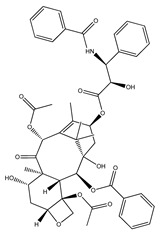	<4.0 × 10^−6 b^	[Ch][Gly]	22.34 ^b^	[83]
			[Ch][Ala]	18.52 ^b^	
			[Ch][Pro]	16.16 ^b^	
			[Ch][Phe]	14.15 ^b^	
			[Ch][Ile]	9.39 ^b^	
			[Ch][Ser]	7.32 ^b^	
			[Ch][Leu]	6.61 ^b^	
Pyrazinecarboxamide	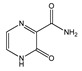	-	[C_2_C_1_im] [NTf_2_]	0.0048 ^a^	[84]
			[C_4_C_1_im] [NTf_2_]	0.0054 ^a^*	
			[C_6_C_1_im] [NTf_2_]	0.0050 ^a^ *	
			[C_8_C_1_im] [NTf_2_]	0.0052 ^a^	
			[C_10_C_1_im] [NTf_2_]	0.0046 ^a^	
			[C_10_C_1_im][CF_3_O_3_S]	0.0116 ^a^	
			[C_2_][NTf_2_]	0.0165 ^a^	[81]
			[P_666(14)_][NTf_2_]	0.0125 ^a^	[72]
Thymoquinone	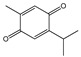	-	[P_666(14)_][NTf_2_]	0.1105 ^a^	[72]

a: molar fraction; b: mg mL^−1^; c: mmol L^−1^; d: mg g^−1^. * Solubility determined at body’s temperature (34–38 °C). If not specified, the reported solubilities were assessed at a temperature range from 21 to 30 °C. Relevant solubilities determined at >38 °C are further addressed in this chapter.

**Table 2:** Antioxidant activity of ILs and comparison with reference compounds.

IL	DPPH Free Radical Scavenging (µM)	Reference Compound	DPPH Free Radical Scavenging (µM)	Reference
2-(methylamino)ethanol ferulate	17.40	Ferulic acid	21.40	[124]
2-(propylamino)ethanol ferulate	16.61			
2-(butylamino)ethanol ferulate	16.34			
3-dimethylamino-1-propanol ferulate	12.93			
3-diethylamino-1-propanol ferulate	14.09			
Bis(ammonium) protocatechuate	5.06–5.98	Protocatechuic acid	15.83	[123]
Cholinium caffeate	2.55	Caffeic acid	1.99	[122]
Cholinium syringate	2.44	Syringic acid	2.04	[122]
Cholinium vanillate	16.03	Vanillic acid	80.46	[122]
Dicholinium ellagate	1.22	Ellagic acid	0.79	[122]

**Table 3:** IL application in topical and transdermal strategies.

Strategy	IL	IL Role	API	Reference
Micellar system	[C_14_C_1_im]Br	Surfactant	Dopamine hydrochloride Acetylcholine chloride	[196]
Micellar system	[C_12_C_1_im]Cl	Surfactant	Ibuprofen	[87]
Micellar system	[C_12_C_1_im]Cl [C_14_C_1_im]Cl	Surfactant	Lidocaine hydrochloride	[197]
Microemulsion	[C_6_C_1_im]Cl [C_4_C_1_im][PF_6_]	Aqueous/Oil phase	Reichardt’s dye (drug model)	[29]
Microemulsion	[C_4_C_1_im][PF_6_]	Oil phase	Etodolac	[79]
Microemulsion	[C_1_C_1_im][(CH_3_O)_2_PO_2_]	Aqueous phase	Acyclovir	[198]
Microemulsion	[Ch][formate] [Ch][lactate] [Ch][propionate] [Ch][oleate]	Non-aqueous phase; Surfactant in oil phase	Acyclovir	[199]
Microemulsion	[C_1_C_1_im][(CH_3_O)_2_PO_2_]	Aqueous phase	Methotrexate	[200]
Microemulsion	[C_2_OHC_1_]Cl [C_1_C_1_im][C_12_SO_3_]	Aqueous phase; Surfactant phase	Dencichine	[201]
Bacterial nanocellulose membranes	[Ch][Caf] [Ch][Gal]	API	Caffeic acid Gallic acid	[202]
Bacterial nanocellulose membranes	[Ch][Ibu] [Ch][Nap] [Ch][Ket]	API	Ibuprofen Naproxen Ketoprofen	[203]
Bacterial nanocellulose membranes	[Ch][B_3_] [Ch][B_5_] [Ch][B_6_]	API	Niacin Pantothenic acid Pyridoxine	[204]
Patch	[Lid][Eto]	Dual API	Lidocaine Etodolac	[205]
Polyvinvylidene fluoride membrane	[Lid][Nap] [Lid][Ibu] [Lid][Dicl]	Dual API	Naproxen Ibuprofen Diclofenac	[206]
PLGA nanoparticles	[Ch][Phe] [Ch][Glu]	API solubilization	Rutin	[207]
Permeation enhancers	[C_8_im]Cl [C_1_C_8_im]Cl [C_1_C_1_C_8_im]Cl	Membrane disruption	Testosterone	[208]
Permeation enhancers	[ProOEt][Ibu]	API	Ibuprofen	[209]
Permeation enhancers	[mPEG_3_N_444_][Sal] [HN_444_][Sal] [Ch][Sal] [C_1_Pyrr][Sal]	API	Salicylic acid	[210]
Permeation enhancers	[Ch][geranate_2_(H)]	API	Geranic acid	[211]

## Abbreviations

**General**	
3-CPP	3-chloro-1-phenyl-1-propanone
AGB-ILs	Ionic liquids analogues of glycine-betaine
APIs	Active pharmaceutical ingredients
API-ILs	Ionic liquid comprising active pharmaceutical ingredients
BC	Bacterial cellulose
Cellulose-g-PLLA	Cellulose-graft-poly (L-lactide)
CrEL	Ethoxylated castor oil
DHQ	2,3-Dihydroquinazolin-4(1H)-one
DMAP	4-Dimethylaminopyridine
DMF	Dimethylformamide
DMSO	Dimethyl sulfoxide
DPPH	2,2-diphenyl-1-picrylhydrazyl
FTIR–ATR	Fourier transform infrared spectroscopy—attenuated total reflectance
HSal	Salicylic acid (neutral form)
IC_50_	Half maximal inhibitory concentration
IL	Ionic liquid
IL/O	IL-in-oil
IL/w	IL-in-water
LCOS	Linoleic acid-grafted chitosan oligosaccharide
LFER	Linear free energy relationship
MIC	Minimal inhibitory concentration
MBC	Minimal biocidal concentration
NSAID	Non-steroidal anti-inflammatory
[Na][Sal]	Sodium salicylate
O/IL	Oil-in-IL
PC12	Pheochromocytoma cells
PLGA	Poly (lactic-co-glycolic acid)
QSAR	Quantitative structure-activity relationship
SAIL	Surface-active ionic liquid
(S)-CPPO	(S)-3-chloro-1-phenyl-1-propanol
SEDDs	Self-emulsifying drug delivery systems
TTAB	Tetradecyltrimethylammonium bromide
W/IL	Water-in-IL
**Ionic Liquids**	
Ammonium	
[C_4_C_1_im][BF_4_]	1-butyl-3-methylimidazolium tetrafluoroborate
[(C_4_C_1_C_1_m][BF_4_]	1-butyl-2,3dimethylimidazolium tetrafluoroborate
[C_6_C_1_im][BF_4_]	1-hexyl-3-methylimidazolium tetrafluoroborate
[C_8_C_1_im][BF_4_]	1-octyl-3-methylimidazolium tetrafluoroborate
[(CH_2_CH=C_2_)C_2_m][BF_4_]	1-allyl-3-ethylimidazolium tetrafluoroborate
[C_4_C_1_im]Br	1-butyl-3-methylimidazolium bromide
[C_14_C_1_im]Br	1-methyl-3-tetradecylimidazolium bromide
[C_8_im]Cl	N-octylimidazolium chloride
[C_1_C_8_im]Cl	1-dodecyl-3-methylimidazolium chloride
[C_1_C_1_C_8_im]Cl	1-methyl-3-tetradecylimidazolium chloride
[C_6_C_1_im]Cl	1-hexyl-3-methylimidazolium chloride
[C_8_C_1_im]Cl	3-methyl-1-octylimidazolium chloride
[C_12_C_1_im]Cl	1-hexadecyl-3-methylimidazolium chloride
[C_14_C_1_im]Cl	1-octyl-3-methyl imidazolium chloride
[(CH_2_CH=C_2_)]Cl	1-allyl-3-methylimidazolium chloride
[C_2_C_1_im][CH_3_COO]	1-ethyl-3-methylimidazolium acetate
[C_2_C_1_im][CH_3_OHPO_2_]	1-ethyl-3-methylimidazolium methylphosphonate
[C_1_C_1_im][(CH_3_O)_2_PO_2_]	Dimethylimidazolium dimethylphosphate
[C_4_C_1_im][C_8_OSO_3_]	1-butyl-3-methylimidazolium octylsulfate
[C_1_C_1_im][C_12_SO_3_]	Dimethylimidazolium dodecanesulfate
[C_2_C_1_im][EtSO_4_]	1-ethyl-3-methylimidazolium ethylsulfate
[C_4_C_1_im][N(CN)_2_]	1-butyl-3-methylimidazolium dicyanamide
[C_2_][NTf_2_]	N-ethyl-2-hydroxy-N,N-dimethylethanammonium bis(trifluoromethylsulfonyl)amide)
[C_2_C_1_im][NTf_2_]	1-ethyl-3-methylimidazolium bis(trifluoromethanesulfonyl)imide
[C_4_C_1_im][NTf_2_]	1-butyl-3-methylimidazolium bis(trifluoromethylsulfonyl)imide
[C_6_C_1_im][NTf_2_]	1-hexyl-3-methylimidazolium bis(trifluoromethanesulfonyl)imide
[C_10_C_1_im][NTf_2_]	1-decyl-3-methylimidazolium bis(trifluoromethanesulfonyl)imide
[C_2_C_1_im][CF_3_O_3_S]	1-ethyl-3-methylimidazolium trifluoromethanesulfonate
[C_4_C_1_im][CF_3_O_3_S]	1-butyl-3-methylimidazolium trifluoromethanesulfonate
[C_10_C_1_im][CF_3_O_3_S]	1-decyl-3-methylimidazolium trifluoromethanesulfonate
[C_4_C_1_im][PF_6_]	1-butyl-3-methylimidazolium hexafluorophosphate
[C_6_C_1_im][PF_6_]	1-hexyl-3-methylimidazolium hexafluorophosphate
[C_8_C_1_im][PF_6_]	1-octyl-3-methylimidazolium hexafluorophosphate
[C_4_C_1_im][SCN])	1-butyl-3-methylimidazolium thiocyanate
[(C₂)₃NC₂]Br	Triethyl[2-ethoxy-2-oxoethyl]ammonium bromide
[C_4_NH_3_][CH_3_COO]	N-butylammonium acetate
[C_6_NH_3_][CH_3_COO]	N-hexylammonium acetate
[C_8_NH_3_][CH_3_COO]	N-octylammonium acetate
[C_4_NH_3_][oleate]	N-butylammonium oleate
[C_6_NH_3_][oleate]	N-hexylammonium oleate
[C_8_NH_3_][oleate]	N-octylammonium oleate
[(C_1_OC_2_)C_1_im][MsO]	1-methoxyethyl-3-methylimidazolium methanesulfonate
[C_2_OHC_1_im]Cl	1-(2-hydroxyethyl)-3-methylimidazolium chloride
[DDA][NO_3_]	Didecyldimethylammonium nitrate
[N_4,1,1,1_][NTf_2_]	N-trimethyl-N-butylammonium bis(trifluoromethanesulfonyl)imide
Cholinium	
[Ch][Ala]	Cholinium alaninate
[Ch][Ile]	Cholinium isoleucine
[Ch][geranate_2_(H)]	Cholinium geranate
[Ch][Glu]	Cholinium L-glutaminate
[Ch][Gly]	Cholinium glycinate
[Ch][Leu]	Cholinium leucinate
[Ch][Phe]	Cholinium phenylalanine
[Ch][Pro]	Cholinium prolinate
[Ch][Se]	Cholinium serinate
[Ch][Try]	Cholinium tryptophan
Morpholinium	
[Nbmd][OH]	4,4′-(butane-1,4-diyl)bis(4-dodecyl-morpholin-4-ium)hydroxide
Phosphonium	
[P_444(14)_]Cl	Tributyltetradecylphosphonium chloride
[P_6,6,6,14_]Cl	Trihexyltetradecylphosphonium chloride
[P_6,6,6,14_][NTf_2_]	Trihexyltetradecylphosphonium bis(trifluoromethylsulfonyl)imide
Pyridinium	
[C_4_C_1_py][DCI]	1-butyl-3-methylpyridinium dichloroiodate
[C_6_C_6_OCOpy][N(CN)_2_]	1-hexyl-3-hexyloxycarbonylpyridinium dicyanamide
[C_6_C_6_OCOpy][NTf_2_]	1-hexyl-3-hexyloxycarbonylpyridinium bis(trifluoromethylsulfonyl)imide
Pyrrolidinium	
[Pyrr_4,1_][NTf_2_]	Butylmethylpyrrolidinium bis(trifluorosulfonyl)amide
**API-ILs**	
[C_4_C_1_im][Ibu]	1-butyl-3-methylimidazolium ibuprofenate
[(C_10_)_2_C_1_C_1_im][Doc]	Didecyidimethylammonium docusate
[(C_10_)_2_C_1_C_1_im][[Ibu]	Didecyldimethylammonium ibuprofenate
[(C_10_)_2_C_1_C_1_im][Pen G]	Didecyldimethylammonium penicillin G
[(C_10_)_2_C_1_C_1_im][Sal]	Didecyldimethylammonium salicylate
[Ch][Amp]	Cholinium ampicilate
[Ch][BA]	Cholinium betulinate
[Ch][B3]	Cholinium nicotinate
[Ch][B5]	Cholinium pantothenate
[Ch][B6]	Cholinium pyridoxylate
[Ch][Caf]	Cholinium caffeate
[Ch][Gal]	Cholinium gallate
[Ch][Ibu]	Cholinium ibuprofenate
[Ch][Ket]	Cholonium ketoprofen
[Ch][Nal]	Cholinium nalixidixate
[Ch][Nap]	Cholinium naproxen
[Ch][Nif]	Cholinium niflumate
[Ch][Sal]	Cholinium salicylate
[Ch]_2_[Ell]	Dicholinium ellagate
[C_2_OHC_1_im][Amp]	1-hydroxy-ethyl-3-methylimidazolium ampicilate
[C_2_OHC_1_im][Ibu]	1-ethanol-3-methylimidazolium ibuprofenate
[C_1_Pyrr][Sal]	1-methylpyrrolidinium salicylate
[HN_444_][Sal]	Tributylammonium salicylate
[Lid][Asp]	Lidocainium acetylsalicylate
[Lid][Dicl]	Lidocainium diclofenac
[Lid][Doc]	Lidocainium docusate
[Lid][Eto]	Lidocainium etodolac
[Lid][Ibu]	Lidocainium ibuprofenate
[Lid][Nap]	Lidocainium naproxenum
[Ran][Doc]	Ranitidine docusate
[mPEG_3_N_444_][Sal]	Triethylene glycol monomethyl ether tributylammonium salicylate
[P_4444_][Ibu]	Tetrabutylphosphonium ibuprofenate
[P_666(14)_][Amp]	Trihexyl-tetradecyl phosphonium ampicilate
[PBu_4_][Sal]_n_H_m-1_	Tetrabutylphosphonium salicylates
[ProOEt][Ibu]	Ethylester ibuprofenate
